# Combining DOE With Neurofuzzy Logic for Healthy Mineral Nutrition of Pistachio Rootstocks *in vitro* Culture

**DOI:** 10.3389/fpls.2018.01474

**Published:** 2018-10-15

**Authors:** Esmaeil Nezami-Alanagh, Ghasem-Ali Garoosi, Mariana Landín, Pedro Pablo Gallego

**Affiliations:** ^1^Applied Plant and Soil Biology, Faculty of Biology, University of Vigo, Vigo, Spain; ^2^Department of Biotechnology, Faculty of Agriculture and Natural Resources, Imam Khomeini International University, Qazvin, Iran; ^3^Department of Pharmacology, Pharmacy and Pharmaceutical Technology, Faculty of Pharmacy, University of Santiago de Compostela, Santiago de Compostela, Spain

**Keywords:** DOE, micropropagation, health shoots, neurofuzzy logic, pistachio rootstock, physiological disorders

## Abstract

The aim of this study was to determine the effects of Murashige and Skoog (MS) salts on optimal growth of two pistachio rootstocks, *P. vera* cv. “Ghazvini” and “UCB1” using design of experiments (DOE) and artificial intelligence (AI) tools. MS medium with 14 macro—and micro-elements was used as base point and its concentration varied from 0 to 5 × MS concentrations. Design of experiments (DOE) software was used to generate a five-dimensional design space by categorizing MS salts into five independent factors (NH_4_NO_3_, KNO_3_, mesos, micros and iron), reducing the experimental design space from 3,125 to just 29 treatments. Typical plant growth parameters such as shoot quality (SQ), proliferation rate (PR), shoot length (SL), and some physiological disorders including shoot-tip necrosis (STN) and callus formation at the base of explants (BC) were evaluated for each treatment. The results were successfully modeled using neurofuzzy logic software. The model delivered new insights, by different sets of “IF–THEN” rules, pinpointing the key role of some ion interactions (${\text{SO}}_{4}^{2-}$ × Cl^−^, K^+^ × ${\text{SO}}_{4}^{2-}$ × EDTA^−^, and Fe^2+^ × Cu^2+^ × ${\text{NO}}_{3}^{-}$) for SQ, PR, and SL, whilst physiological disorders (STN and BC) were governed mainly by independent ions as Fe^2+^ and EDTA^−^, respectively. In our opinion, the methodology and results obtained in this study is extremely useful to understand the effect of mineral nutrients on pistachio *in vitro* culture, through discovering new complex interactions among macro—and micro-elements which can be implemented to design new media of plant tissue culture and improve healthy plant micropropagation for any plant species.

## Introduction

Most authors recommend the use of MS as a basal culture medium for the micropropagation for different species of *Pistacia* as *P. vera, P. khinjuk, P. lenthiskus, P. atlantica* (Barghchi and Alderson, 1983a,b, 1985; Yang and Lüdders, 1993; Benmahioul et al., 2012; Akdemir et al., 2014). Other media specifically design for woody plants, as DKW (Driver Kuniyuki Walnut medium; Driver and Kuniyuki, 1984) or WPM (Woody Plant Medium; Lloyd and McCown, 1980) for *P. vera* L. (Gonzalez and Frutos, 1990; Benmahioul et al., 2009) have also been applied. However, the use of these standard media increases the cost of micropropagation of *Pistacia sp*. due to the appearance of physiological disorders, such as shoot tip necrosis (STN), hyperhydricity or the formation of callus (BC) at the base of the shoots (Abousalim and Mantell, 1992, 1994; Dolcet-Sanjuan and Claveria, 1995; Onay, 1996).

To avoid those problems, different alternatives have been suggested, based mainly on the combination of macro-, micro-elements and/or vitamins previously proposed for improving the effectiveness of the multiplication of shoots (Onay, 2000; Tilkat et al., 2005, 2008; Benmahioul et al., 2012), or on the incorporation of additional ingredients to the basal culture media, such as calcium gluconate, silver nitrate, ascorbic acid or vanillin, among others (Abousalim and Mantell, 1992, 1994; Parfitt and Almehdi, 1994; Dolcet-Sanjuan and Claveria, 1995; Mederos-Molina and Trujillo, 1999; Akdemir et al., 2012; Kilinç et al., 2015; Marín et al., 2017).

The establishment of an adequate design space should allow to improve the understanding of the role of mineral nutrition in response of pistachio shoots and, ultimately, to determine a culture medium suitable for mass micropropagation of *Pistacia*.

The design of experiments (DOE) provides researchers to program investigations by dramatically reducing the number of combinations to be studied and distributing them appropriately in the *n*-dimensional design space (Niedz and Evens, 2016). As a result, it is possible to obtain general conclusions, from the generated databases under statistically optimal conditions. As an example, a five-dimensional design space has been constructed on the basis of MS medium composition using a DOE software in order to optimize mineral nutrition of different species as *Citrus sinenis* (Niedz and Evens, 2007); the hybrid Gerbera (Niedz et al., 2014), *Rubus idaeus L*. (Poothong and Reed, 2014, 2015), *Corylus avellana L*. (Hand et al., 2014; Akin et al., 2017a,b), Pear sp. (Reed et al., 2016) and *Prunus armeniaca* Lam (Kovalchuk et al., 2017). As mineral nutrients of culture medium includes many components, the five-dimensional design space proposed elsewhere (Reed et al., 2016) was carried out by dividing all the mineral nutrients of MS into five factors, allowing to reduce the number of treatments from 3,125 (5^5^ combinations treatments) to just 29 treatments.

If we think that mineral nutrients are just a part of the plant tissue culture medium and that other components such as vitamins or plant growth regulators need also being added and optimized, it is clear that finding adequate composition for plant tissue culture media requires to deal with high dimensional spaces, which represent a great challenge for researchers (Niedz and Evens, 2016). In this situation, artificial intelligence tools are presented as promising candidates to extract information from the big databases that can be generated during the development of the culture media using high dimensional spaces (Gallego et al., 2011). Several studies have illustrated the effectiveness of artificial neural networks (ANNs) (Gago et al., 2010a,b,c,d, 2014) and hybrid systems as neurofuzzy logic (Gallego et al., 2011; Nezami-Alanagh et al., 2014; Ayuso et al., 2017) in modeling and extracting information from experiments carried out even without a well define design space. Even more, using those artificial intelligence tools, it has been identified the negative effect of some additional ingredients that had been proposed for improving pistachio culture, especially the silver and the gluconate ions (Nezami-Alanagh et al., 2017).

The objective of this research was to combine DOE and an artificial intelligence tool, neurofuzzy logic, first to establish a well experimental design space and second to determine the key mineral nutrients in pistachio culture. The use of two different pistachio rootstocks, “Ghazvini” and “UCB1,” will make it possible to draw conclusions about the influence of genotype variations on the nutritional needs of culture.

## Materials and methods

### Plant materials and *in vitro* culture conditions

*Pistacia vera* cv. “Ghazvini,” a high tolerant to salinity rootstock (Karimi et al., 2009) and”UCB1”(interspecific hybrid *P. atlantica* × *P. integrima*), a vigorous rootstock that has resistance to wilt by *Werticillium*, were micropropagated on MS medium (Murashige and Skoog, 1962) supplemented with 1.1 mg L^−1^ N^6^-benzyladenine (BAP), 0.1 mg l^−1^ indole-3-butyric acid (IBA), 30 g L^−1^ sucrose and 5.7 g L^−1^ agar. The pH was adjusted to 5.7 prior autoclaving (121°C, 1 kg cm^−2^ s^−1^ for 20 min). The cultures were kept under 16 h photoperiod (white fluorescent tubes; irradiance of 65 μmol m^−2^ s^−1^) and day/night temperature of 25/20 ± 2°C and subcultured into a fresh medium every 30 days. Pistachio explants, with almost the same shoot length (~1 cm) and containing 1–2 axillary buds, were randomly selected just before placing in glass boxes (180 ml) containing 25 ml on the set of treatment combinations. After 3 subcultures (30 days interval) on the same culture media, growth parameters and physiological disorders were evaluated as follows: shoot quality (SQ; scored as 1 = necrotic, 2 = poor, 3 = moderate, 4 = good), proliferation rate (PR; number of new regenerated shoots per explant), shoot length (SL; length of new regenerated shoots per explant in cm), shoot-tip necrosis (STN; scored as 1 = none, 2 = very low, 3 = moderate, 4 = high), and basal callus (BC; callus formation at the cut edge of shoots in grams).

Each treatment consisted of two replicates glass boxes (180 ml) sealed with crystal caps, containing five explants each. The experiments were carried out in triplicate.

### Design of experiment (DOE) and data acquisition

For the modification of MS medium, the 14 salts of MS basal medium were subdivided into five independent factors including: (i) NH_4_NO_3_, (ii) KNO_3_, (iii) mesos, (iv) micros, and (v) iron, over a range of concentrations expressed as × MS levels (Table 1).

**Table 1:** The five mineral nutrient factors used to construct the experimental design space based on MS salts, and concentration range expressed as × MS levels.

**Factors**	**MS salts**	**Range**
Factor1	KNO_3_	0.0–1.0×
Factor 2	NH_4_NO_3_	0.2–1.1×
Factor 3 (Mesos)	CaCl_2_.2H_2_O KH_2_PO_4_ MgSO4.7H2O	0.25–3.0×
Factor 4 (Micros)	MnSO_4_.4H_2_O ZnSO_4_.7H_2_O CuSO_4_.5H_2_O KI CoCl_2_.6H_2_O H_3_BO_3_ Na_2_MoO_4_.2H_2_O	0.1–4.0×
Factor 5 (Iron)	FeSO_4_.7H_2_O Na_2_EDTA.2H_2_O	1.0–5.0×

The five-factor experimental design was a 28-model-point using IV-optimal response surface using software application Design-Expert®8 (Design-Expert, 2010) plus another point with MS salt concentration as control (Table 2).

**Table 2:** Five factor design including 29 treatment points based on × MS concentrations of mineral nutrients.

**Treatments**	**Factor 1 KNO_3_**	**Factor 2 NH_4_NO_3_**	**Factor 3 Mesos**	**Factor 4 Minors**	**Factor 5 Iron**
#1	1.00	0.20	0.25	4.00	2.33
#2	0.00	0.20	2.08	4.00	1.00
#3	1.00	1.10	2.08	0.10	1.00
#4	0.00	1.10	0.25	1.40	3.66
#5	0.00	1.10	3.00	4.00	1.00
#6	0.50	0.20	3.00	0.10	5.00
#7	0.00	0.20	0.25	0.10	1.00
#8	0.00	1.10	3.00	4.00	5.00
#9	0.33	0.50	0.25	0.10	5.00
#10	0.00	0.50	3.00	0.10	2.33
#11	1.00	1.10	0.25	4.00	1.00
#12	0.00	0.20	1.16	2.70	5.00
#13	1.00	0.65	0.25	0.10	1.00
#14	0.33	0.20	3.00	4.00	3.66
#15	1.00	0.20	1.16	0.10	3.66
#16	0.66	1.10	3.00	0.10	3.66
#17	0.00	1.10	1.62	0.10	1.00
#18	1.00	0.80	3.00	4.00	2.33
#19	1.00	0.80	0.25	2.70	5.00
#20	1.00	1.10	0.25	0.10	3.66
#21	0.50	0.65	1.62	2.05	3.00
#22	1.00	1.10	3.00	2.70	5.00
#23	1.00	0.20	3.00	2.70	5.00
#24	0.50	0.65	1.62	2.05	2.00
#25	0.66	0.20	3.00	1.40	1.00
#26	0.00	0.80	3.00	1.40	5.00
#27	0.00	0.80	0.25	4.00	2.33
#28	0.33	1.10	1.16	4.00	5.00
Control (MS)	1.00	1.00	1.00	1.00	1.00

### Modeling tools

The database was modeled using commercial software (FormRules® 4.03, Intelligensys Ltd., UK) that combines artificial neural networks (ANNs) and fuzzy logic tools. A detailed description of the software package has reported elsewhere (Shao et al., 2006). During the training process 24 variables were included. As *inputs*, 19 independent variables or factors including genotype (“UCB1” and “Ghazvini” rootstocks) and 18 ions (${\text{NH}}_{4}^{+}$, ${\text{NO}}_{3}^{-}$, K^+^, Ca^2+^, Mg^2+^, ${\text{PO}}_{4}^{2-}$, ${\text{SO}}_{4}^{2-}$, Cl^−^, Fe^2+^, ${\text{BO}}_{3}^{-}$, Mn^2+^, Zn^2+^, Cu^2+^, ${\text{MoO}}_{2}^{2-}$, Na^+^, Co^2+^, I^−^ and EDTA^−^) and as *outputs*, five dependent variables or parameters (SQ, PR, SL, STN, and BC mean responses of 10 shoots per treatment for each genotype).

Modeling process was carried out as previously described by Nezami-Alanagh et al. (2017). Individual models were developed for each *output*, the predictability of which was tested using the Train Set *R*^2^ value or coefficient of determination expressed as percentage, which is indicative of the percentage of variation of an *output* that is explained by the *inputs* in the model, it is defined by equation 1.

(1) $$\begin{array}{l} R^{2}=\left(1-\frac{{\overset{n}{\underset{i=1}{\sum }}\left(y_{i}-y_{i}^{\prime }\right)}^{2}}{\overset{n}{\underset{i=1}{\sum }}{\left(y_{i}-y_{i}^{\prime \prime }\right)}^{2}}\right)\times 100\% \end{array}$$

Where *y* is the experimental point in the data set, *y*′ is the predicted value calculated by the model and *y*″ is the mean of the dependent variable. The larger the value of the train set *R*^2^, the more the model captured the variation in the training data. Values of *R*^2^ between 70 and 99.9% are considered indicative of good model predictabilities (Colbourn and Rowe, 2005, 2009).

The Analysis of Variance (ANOVA) was used to evaluate the differences between experimental outputs and predicted by the model ones. *F* ratios higher than the critical *f* for the degrees of freedom of the model indicate that there are not significant differences between predicted by the model and the experimental *outputs*, and therefore, that the models are accurate. Models were developed using the default parameters in the software as shown in Table 3.

**Table 3:** The training parameters setting with neurofuzzy logic.

**Critical factors for neurofuzzy logic model**
*Minimization parameters*
Ridge regression factor:1e^−6^
*Model selection criteria*
Structural risk minimization (SRM)
C1 = 0.8–0.916; C2 = 4.8
Number of set densities:2
Set densities: 2, 3
Adapt *nodes*: True
Max.*Inputs* per SubModel: 4
Max. *nodes* per input: 15

To carry out the modeling, avoiding ion confounding problem pointed out by Niedz and coworkers (Niedz and Evens, 2006), the ionic composition of each treatment was calculated from its corresponding macro—and micro-elements (Tables 4 and S1).

**Table 4:** Ion compositions of the different culture media based on the five-factor design space to optimize micropropagation of pistachio rootstocks and average results for each parameter.

**Treatment**	**Genotype**	**Ions (mM)**																		**Growth parameters**			**Physiological disorders**	
		**${\text{NH}}_{4}^{+}$**	**${\text{NO}}_{3}^{-}$**	**K^+^**	**Ca^2+^**	**Mg^2+^**	**${\text{PO}}_{4}^{2-}$**	**${\text{SO}}_{4}^{2-}$**	**Cl^−^**	**Fe^2+^**	**${\text{BO}}_{3}^{-}$**	**Mn^2+^**	**Zn^2+^**	**Cu^2+^**	**${\text{MoO}}_{2}^{2-}$**	**Na^+^**	**Co^2+^**	**I^−^**	**EDTA^−^**	**SQ**	**PR**	**SL (cm)**	**STN**	**BC (g)**
#1	UCB1	4.12	22.92	19.13	0.75	0.38	0.31	1.13	1.50	0.23	0.40	0.40	0.12	0.000401	0.004	0.48	0.00042029	0.02	0.23	1.89 ± 0.16	2.40 ± 0.40	0.74 ± 0.07	1.88 ± 0.18	0.128 ± 0.010
#2	UCB1	4.12	4.12	2.62	6.24	3.13	2.60	3.75	12.47	0.10	0.40	0.40	0.12	0.000401	0.004	0.21	0.00042029	0.02	0.10	2.10 ± 0.13	2.85 ± 0.28	0.62 ± 004	1.97 ± 0.27	0.194 ± 0.017
#3	UCB1	22.68	41.47	21.40	6.24	3.13	2.60	3.24	12.47	0.10	0.01	0.01	0.00	1E-05	0.000	0.20	1.05E-05	0.0005	0.10	2.73 ± 0.13	2.50 ± 0.19	0.77 ± 0.05	1.05 ± 0.05	0.225 ± 0.016
#4	UCB1	22.68	22.68	0.32	0.75	0.38	0.31	0.93	1.50	0.37	0.14	0.14	0.04	0.00014	0.001	0.74	0.0001471	0.007	0.37	1.72 ± 0.16	1.56 ± 0.29	0.81 ± 0.09	2.61 ± 0.32	0.006 ± 0.002
#5	UCB1	22.68	22.68	3.77	8.98	4.50	3.75	5.12	17.96	0.10	0.40	0.40	0.12	0.000401	0.004	0.21	0.00042029	0.02	0.10	3.00 ± 0.07	3.20 ± 0.38	1.35 ± 0.15	1.05 ± 0.05	0.245 ± 0.029
#6	UCB1	4.12	13.52	13.14	8.98	4.50	3.75	5.02	17.96	0.50	0.01	0.01	0.00	1E-05	0.000	1.00	1.05E-05	0.0005	0.50	1.00 ± 0.00	0	0	4.00 ± 0.00	0
#7	UCB1	4.12	4.12	0.31	0.75	0.38	0.31	0.49	1.50	0.10	0.01	0.01	0.00	1E-05	0.000	0.20	1.05E-05	0.0005	0.10	2.65 ± 0.18	3.00 ± 0.39	0.51 ± 0.06	1.00 ± 0.00	0.059 ± 0.014
#8	UCB1	22.68	22.68	3.77	8.98	4.50	3.75	5.53	17.96	0.50	0.40	0.40	0.12	0.000401	0.004	1.01	0.00042029	0.02	0.50	2.00 ± 0.22	2.90 ± 0.31	0.57 ± 0.08	2.25 ± 0.35	0.074 ± 0.010
#9	UCB1	10.31	16.57	6.58	0.75	0.38	0.31	0.89	1.50	0.50	0.01	0.01	0.00	1E-05	0.000	1.00	1.05E-05	0.0005	0.50	1.00 ± 0.00	0	0	4.00 ± 0.00	0
#10	UCB1	10.31	10.31	3.75	8.98	4.50	3.75	4.75	17.96	0.23	0.01	0.01	0.00	1E-05	0.000	0.47	1.05E-05	0.0005	0.23	2.44 ± 0.29	3.89 ± 0.35	0.45 ± 0.05	1.55 ± 0.29	0.143 ± 0.016
#11	UCB1	22.68	41.47	19.13	0.75	0.38	0.31	1.00	1.50	0.10	0.40	0.40	0.12	0.000401	0.004	0.21	0.00042029	0.02	0.10	1.40 ± 0.16	2.30 ± 0.47	0.62 ± 0.11	1.60 ± 0.30	0.112 ± 0.022
#12	UCB1	4.12	4.12	1.47	3.49	1.75	1.46	2.60	6.98	0.50	0.27	0.27	0.08	0.00027	0.003	1.01	0.0002837	0.0135	0.50	2.28 ± 0.27	2.44 ± 0.24	0.68 ± 0.07	1.77 ± 0.32	0.040 ± 0.006
#13	UCB1	13.40	32.19	19.11	0.75	0.38	0.31	0.49	1.50	0.10	0.01	0.01	0.00	1E-05	0.000	0.20	1.05E-05	0.0005	0.10	1.94 ± 0.05	2.89 ± 0.35	0.46 ± 0.04	3.05 ± 0.21	0.120 ± 0.008
#14	UCB1	4.12	10.39	10.03	8.98	4.50	3.75	5.39	17.96	0.37	0.40	0.40	0.12	0.000401	0.004	0.74	0.00042029	0.02	0.37	2.39 ± 0.13	3.11 ± 0.26	0.62 ± 0.07	1.22 ± 0.22	0.184 ± 0.019
#15	UCB1	4.12	22.92	20.25	3.49	1.75	1.46	2.13	6.98	0.37	0.01	0.01	0.00	1E-05	0.000	0.73	1.05E-05	0.0005	0.37	1.63 ± 0.21	2.05 ± 0.22	0.62 ± 0.07	2.67 ± 0.32	0.079 ± 0.005
#16	UCB1	22.68	35.20	16.28	8.98	4.50	3.75	4.88	17.96	0.37	0.01	0.01	0.00	1E-05	0.000	0.73	1.05E-05	0.0005	0.37	2.19 ± 0.33	2.50 ± 0.37	0.56 ± 0.08	1.75 ± 0.41	0.106 ± 0.015
#17	UCB1	22.68	22.68	2.03	4.86	2.44	2.03	2.55	9.73	0.10	0.01	0.01	0.00	1E-05	0.000	0.20	1.05E-05	0.0005	0.10	2.78 ± 0.22	2.78 ± 0.32	0.50 ± 0.05	1.11 ± 0.11	0.249 ± 0.054
#18	UCB1	16.49	35.28	22.56	8.98	4.50	3.75	5.26	17.96	0.23	0.40	0.40	0.12	0.000401	0.004	0.48	0.00042029	0.02	0.23	3.31 ± 0.36	2.50 ± 0.42	1.23 ± 0.21	1.56 ± 0.37	0.190 ± 0.023
#19	UCB1	16.49	35.28	19.12	0.75	0.38	0.31	1.23	1.50	0.50	0.27	0.27	0.08	0.00027	0.003	1.01	0.0002837	0.0135	0.50	1.00 ± 0.00	0	0	4.00 ± 0.00	0
#20	UCB1	22.68	41.47	19.11	0.75	0.38	0.31	0.76	1.50	0.37	0.01	0.01	0.00	1E-05	0.000	0.73	1.05E-05	0.0005	0.37	1.57 ± 0.20	3.00 ± 0.37	0.57 ± 0.10	2.92 ± 0.51	0.056 ± 0.007
#21	UCB1	13.40	22.80	11.44	4.86	2.44	2.03	3.01	9.73	0.30	0.21	0.20	0.06	0.000205	0.002	0.61	0.0002154	0.01025	0.30	3.05 ± 0.17	2.85 ± 0.22	0.88 ± 0.08	1.32 ± 0.16	0.184 ± 0.017
#22	UCB1	22.68	41.47	22.55	8.98	4.50	3.75	5.36	17.96	0.50	0.27	0.27	0.08	0.00027	0.003	1.01	0.0002837	0.0135	0.50	1.00 ± 0.00	0	0	4.00 ± 0.00	0
#23	UCB1	4.12	22.92	22.55	8.98	4.50	3.75	5.36	17.96	0.50	0.27	0.27	0.08	0.00027	0.003	1.01	0.0002837	0.0135	0.50	1.00 ± 0.00	0	0	4.00 ± 0.00	0
#24	UCB1	13.40	22.80	11.44	4.86	2.44	2.03	2.91	9.73	0.20	0.21	0.20	0.06	0.000205	0.002	0.41	0.0002154	0.01025	0.20	3.28 ± 0.21	2.78 ± 0.19	1.15 ± 0.15	1.05 ± 0.05	0.229 ± 0.016
#25	UCB1	4.12	16.65	16.28	8.98	4.50	3.75	4.79	17.96	0.10	0.14	0.14	0.04	0.00014	0.001	0.20	0.0001471	0.007	0.10	2.50 ± 0.13	3.36 ± 0.36	0.71 ± 0.07	1.13 ± 0.13	0.211 ± 0.025
#26	UCB1	16.49	16.49	3.75	8.98	4.50	3.75	5.19	17.96	0.50	0.14	0.14	0.04	0.00014	0.001	1.00	0.0001471	0.007	0.50	2.20 ± 0.21	3.30 ± 0.33	0.45 ± 0.06	1.35 ± 0.23	0.059 ± 0.004
#27	UCB1	16.49	16.49	0.33	0.75	0.38	0.31	1.13	1.50	0.23	0.40	0.40	0.12	0.000401	0.004	0.48	0.00042029	0.02	0.23	2.17 ± 0.27	2.33 ± 0.40	0.61 ± 0.05	2.11 ± 0.45	0.056 ± 0.007
#28	UCB1	22.68	28.94	7.74	3.49	1.75	1.46	2.77	6.98	0.50	0.40	0.40	0.12	0.000401	0.004	1.01	0.00042029	0.02	0.50	2.63 ± 0.20	2.75 ± 0.30	0.70 ± 0.08	1.05 ± 0.80	0.115 ± 0.011
MS (Control)	UCB1	20.61	39.41	20.05	2.99	1.50	1.25	1.73	5.99	0.10	0.10	0.10	0.03	0.0001	0.001	0.20	0.00010507	0.005	0.10	3.00 ± 0.35	2.60 ± 0.50	2.36 ± 0.70	2.10 ± 0.55	0.225 ± 0.030
#1	Ghazvini	4.12	22.92	19.13	0.75	0.38	0.31	1.13	1.50	0.23	0.40	0.40	0.12	0.000401	0.004	0.48	0.00042029	0.02	0.23	2.28 ± 0.14	2.89 ± 0.38	0.75 ± 0.12	1.88 ± 0.18	0.084 ± 0.009
#2	Ghazvini	4.12	4.12	2.62	6.24	3.13	2.60	3.75	12.47	0.10	0.40	0.40	0.12	0.000401	0.004	0.21	0.00042029	0.02	0.10	2.62 ± 0.12	3.45 ± 0.24	0.60 ± 0.02	1.00 ± 0.00	0.191 ± 0.012
#3	Ghazvini	22.68	41.47	21.40	6.24	3.13	2.60	3.24	12.47	0.10	0.01	0.01	0.00	1E-05	0.000	0.20	1.05E-05	0.0005	0.10	1.96 ± 0.25	3.18 ± 0.32	0.83 ± 0.07	1.28 ± 0.14	0.187 ± 0.018
#4	Ghazvini	22.68	22.68	0.32	0.75	0.38	0.31	0.93	1.50	0.37	0.14	0.14	0.04	0.00014	0.001	0.74	0.0001471	0.007	0.37	1.25 ± 0.08	2.20 ± 0.24	0.70 ± 0.09	3.65 ± 0.15	0.001 ± 0.001
#5	Ghazvini	22.68	22.68	3.77	8.98	4.50	3.75	5.12	17.96	0.10	0.40	0.40	0.12	0.000401	0.004	0.21	0.00042029	0.02	0.10	3.60 ± 0.14	3.40 ± 0.42	1.36 ± 0.14	1.75 ± 0.38	0.287 ± 0.047
#6	Ghazvini	4.12	13.52	13.14	8.98	4.50	3.75	5.02	17.96	0.50	0.01	0.01	0.00	1E-05	0.000	1.00	1.05E-05	0.0005	0.50	1.00 ± 0.00	0	0	4.00 ± 0.00	0
#7	Ghazvini	4.12	4.12	0.31	0.75	0.38	0.31	0.49	1.50	0.10	0.01	0.01	0.00	1E-05	0.000	0.20	1.05E-05	0.0005	0.10	1.95 ± 0.24	4.10 ± 0.43	0.51 ± 0.04	2.38 ± 0.46	0.033 ± 0.007
#8	Ghazvini	22.68	22.68	3.77	8.98	4.50	3.75	5.53	17.96	0.50	0.40	0.40	0.12	0.000401	0.004	1.01	0.00042029	0.02	0.50	1.89 ± 0.20	3.44 ± 0.05	0.73 ± 0.04	1.88 ± 0.45	0.060 ± 0.017
#9	Ghazvini	10.31	16.57	6.58	0.75	0.38	0.31	0.89	1.50	0.50	0.01	0.01	0.00	1E-05	0.000	1.00	1.05E-05	0.0005	0.50	1.00 ± 0.00	0	0	4.00 ± 0.00	0
#10	Ghazvini	10.31	10.31	3.75	8.98	4.50	3.75	4.75	17.96	0.23	0.01	0.01	0.00	1E-05	0.000	0.47	1.05E-05	0.0005	0.23	2.50 ± 0.17	5.45 ± 0.45	0.52 ± 0.05	1.18 ± 0.18	0.146 ± 0.019
#11	Ghazvini	22.68	41.47	19.13	0.75	0.38	0.31	1.00	1.50	0.10	0.40	0.40	0.12	0.000401	0.004	0.21	0.00042029	0.02	0.10	1.70 ± 0.18	3.00 ± 0.25	0.79 ± 0.08	2.95 ± 0.31	0.102 ± 0.006
#12	Ghazvini	4.12	4.12	1.47	3.49	1.75	1.46	2.60	6.98	0.50	0.27	0.27	0.08	0.00027	0.003	1.01	0.0002837	0.0135	0.50	1.11 ± 0.07	3.33 ± 0.47	0.64 ± 0.03	3.87 ± 0.08	0.026 ± 0.004
#13	Ghazvini	13.40	32.19	19.11	0.75	0.38	0.31	0.49	1.50	0.10	0.01	0.01	0.00	1E-05	0.000	0.20	1.05E-05	0.0005	0.10	1.90 ± 0.20	3.40 ± 0.54	0.81 ± 0.11	2.61 ± 0.37	0.086 ± 0.011
#14	Ghazvini	4.12	10.39	10.03	8.98	4.50	3.75	5.39	17.96	0.37	0.40	0.40	0.12	0.000401	0.004	0.74	0.00042029	0.02	0.37	2.19 ± 0.04	2.63 ± 0.26	0.94 ± 0.08	1.28 ± 0.28	0.076 ± 0.018
#15	Ghazvini	4.12	22.92	20.25	3.49	1.75	1.46	2.13	6.98	0.37	0.01	0.01	0.00	1E-05	0.000	0.73	1.05E-05	0.0005	0.37	1.58 ± 0.16	2.65 ± 0.31	0.67 ± 0.05	2.76 ± 0.32	0.052 ± 0.007
#16	Ghazvini	22.68	35.20	16.28	8.98	4.50	3.75	4.88	17.96	0.37	0.01	0.01	0.00	1E-05	0.000	0.73	1.05E-05	0.0005	0.37	1.56 ± 0.29	1.88 ± 0.22	0.82 ± 0.18	2.91 ± 0.41	0.037 ± 0.005
#17	Ghazvini	22.68	22.68	2.03	4.86	2.44	2.03	2.55	9.73	0.10	0.01	0.01	0.00	1E-05	0.000	0.20	1.05E-05	0.0005	0.10	2.50 ± 0.20	3.00 ± 0.48	0.58 ± 0.06	1.00 ± 0.00	0.227 ± 0.022
#18	Ghazvini	16.49	35.28	22.56	8.98	4.50	3.75	5.26	17.96	0.23	0.40	0.40	0.12	0.000401	0.004	0.48	0.00042029	0.02	0.23	2.80 ± 0.24	3.00 ± 0.29	0.99 ± 0.23	1.35 ± 0.19	0.228 ± 0.048
#19	Ghazvini	16.49	35.28	19.12	0.75	0.38	0.31	1.23	1.50	0.50	0.27	0.27	0.08	0.00027	0.003	1.01	0.0002837	0.0135	0.50	1.00 ± 0.00	0	0	4.00 ± 0.00	0
#20	Ghazvini	22.68	41.47	19.11	0.75	0.38	0.31	0.76	1.50	0.37	0.01	0.01	0.00	1E-05	0.000	0.73	1.05E-05	0.0005	0.37	1.22 ± 0.12	2.00 ± 0.33	0.76 ± 0.10	3.92 ± 0.07	0.023 ± 0.006
#21	Ghazvini	13.40	22.80	11.44	4.86	2.44	2.03	3.01	9.73	0.30	0.21	0.20	0.06	0.000205	0.002	0.61	0.0002154	0.01025	0.30	2.63 ± 0.24	2.80 ± 0.31	1.02 ± 0.09	1.81 ± 0.25	0.088 ± 0.010
#22	Ghazvini	22.68	41.47	22.55	8.98	4.50	3.75	5.36	17.96	0.50	0.27	0.27	0.08	0.00027	0.003	1.01	0.0002837	0.0135	0.50	1.00 ± 0.00	0	0	4.00 ± 0.00	0
#23	Ghazvini	4.12	22.92	22.55	8.98	4.50	3.75	5.36	17.96	0.50	0.27	0.27	0.08	0.00027	0.003	1.01	0.0002837	0.0135	0.50	1.00 ± 0.00	0	0	4.00 ± 0.00	0
#24	Ghazvini	13.40	22.80	11.44	4.86	2.44	2.03	2.91	9.73	0.20	0.21	0.20	0.06	0.000205	0.002	0.41	0.0002154	0.01025	0.20	3.80 ± 0.06	2.55 ± 0.23	1.35 ± 0.11	1.12 ± 0.08	0.182 ± 0.014
#25	Ghazvini	4.12	16.65	16.28	8.98	4.50	3.75	4.79	17.96	0.10	0.14	0.14	0.04	0.00014	0.001	0.20	0.0001471	0.007	0.10	3.14 ± 0.21	2.86 ± 0.63	0.86 ± 0.10	1.14 ± 0.14	0.183 ± 0.007
#26	Ghazvini	16.49	16.49	3.75	8.98	4.50	3.75	5.19	17.96	0.50	0.14	0.14	0.04	0.00014	0.001	1.00	0.0001471	0.007	0.50	1.50 ± 0.21	3.30 ± 0.53	0.59 ± 0.05	2.37 ± 0.53	0.039 ± 0.005
#27	Ghazvini	16.49	16.49	0.33	0.75	0.38	0.31	1.13	1.50	0.23	0.40	0.40	0.12	0.000401	0.004	0.48	0.00042029	0.02	0.23	1.86 ± 0.09	2.55 ± 0.34	0.85 ± 0.12	1.61 ± 0.33	0.039 ± 0.012
#28	Ghazvini	22.68	28.94	7.74	3.49	1.75	1.46	2.77	6.98	0.50	0.40	0.40	0.12	0.000401	0.004	1.01	0.00042029	0.02	0.50	1.30 ± 0.13	2.00 ± 0.25	0.96 ± 0.10	4.00 ± 0.00	0.046 ± 0.008
MS (Control)	Ghazvini	20.61	39.41	20.05	2.99	1.50	1.25	1.73	5.99	0.10	0.10	0.10	0.03	0.0001	0.001	0.20	0.00010507	0.005	0.10	3.17 ± 0.27	3.33 ± 0.42	1.60 ± 0.14	2.83 ± 0.38	0.255 ± 0.021

Among the statistical fitness criteria used by FormRules®, Structural Risk Minimization (SRM) was selected because it allowed obtaining the models with the highest predictabilities together with the simplest and more intelligible rules. FormRules® has been designed on the basis of the ASMOD algorithm (adaptive spline modeling of data) which allows the models to be divided into several submodels to easily generate “IF-THEN” rules and the interpretation of results. The result of neurofuzzy logic technology, as described by Gago et al. (2011), is a predictive model presented as “IF-THEN” rules, together with a degree of membership that varies between 0.00, “Low value,” and 1.00, “High value” (Shao et al., 2006).

## Results

**Figure 1:**
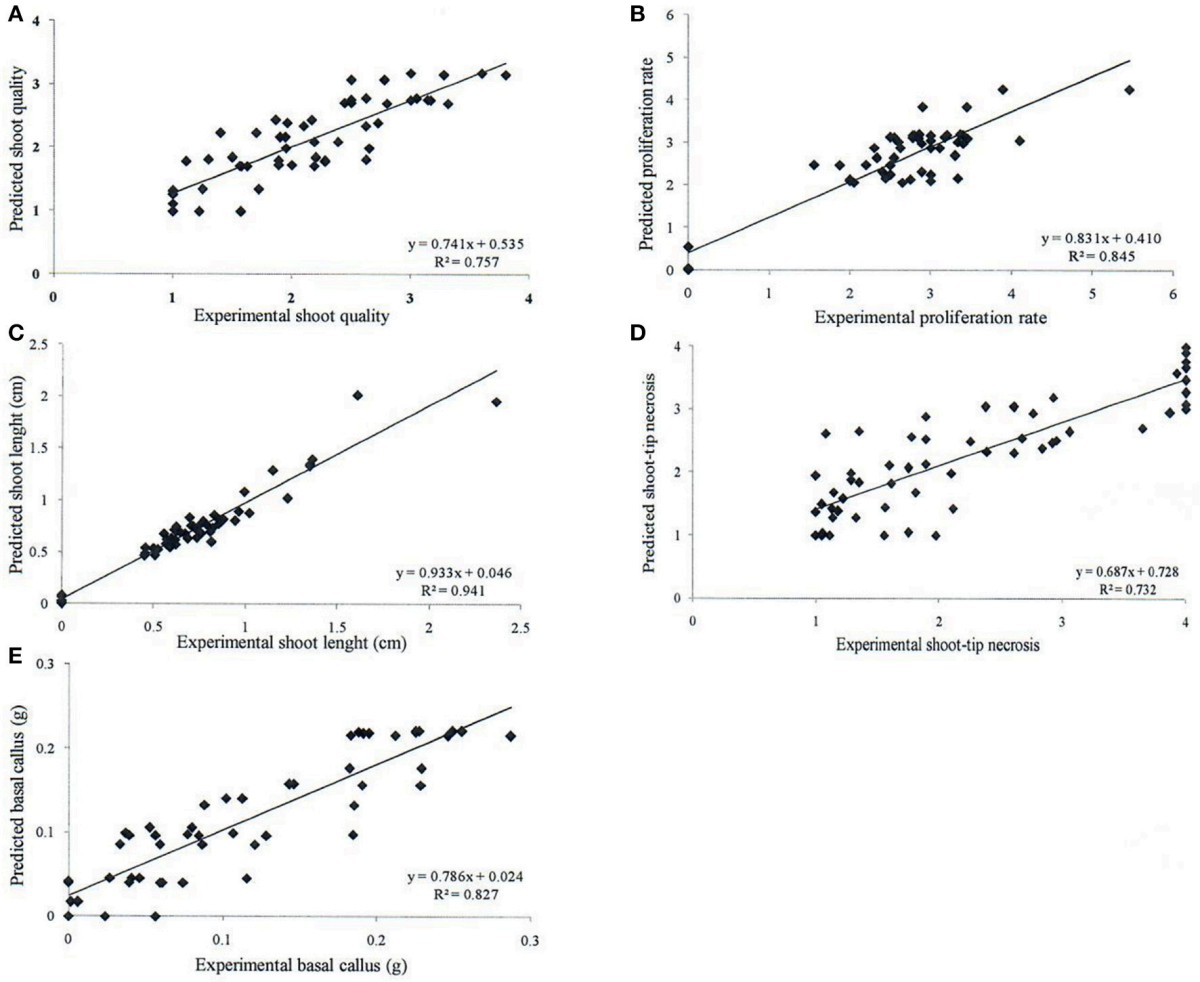
Determination coefficient (*R*^2^) of experimental vs. predicted values achieved by neurofuzzy logic models for the different parameters or *outputs* studied: **(A)** shoot quality, **(B)** proliferation rate, **(C)** shoot length, **(D)** shoot-tip necrosis, and **(E)** basal callus.

Neurofuzzy logic succeeded in simultaneously modeling the five growth parameters studied showing high Train Set *R*^2^ between experimental and predicted values (73.2 ≤ *R*^2^ ≤ 94.1%), which are an indication of high predictability of the algorithm (Table 5 and Figure 1).

**Table 5:** Critical factors from the neurofuzzy logic models, coefficient of determination (train set *R*^2^ in percentage) and ANOVA parameters for training (*F* ratio, degree of freedom (df1: model and df2: total), and *f* critical value for α = 0.01) for each *output*.

***Outputs***	**Submodel**	**Significant *inputs***	**Train Set *R^2^* (%)**	***F* ratio**	**d*f1*, d*f2***	***f* critical**
SQ	1	Fe^2+^	75.7	14.06	10, 47	2.72
	**2**	**${\text{SO}}_{4}^{2-}$×**Cl**^−^**				
	3	K^+^				
	4	${\text{NH}}_{4}^{+}$				
PR	**1**	**K**^+^ × **EDTA**^−^ × ${\text{SO}}_{4}^{2-}$	84.5	18.42	13, 44	2.56
	2	Fe^2+^ × ${\text{BO}}_{3}^{-}$				
SL (cm)	**1**	**Fe**^2+^ × ${\text{NO}}_{3}^{-}$ × **Cu**^2+^	94.1	21.97	24, 33	2.40
	2	K^+^ × Cl^−^				
	3	Genotype				
STN	**1**	**EDTA**^−^	73.2	14.94	8, 49	2.89
	2	${\text{SO}}_{4}^{2-}$				
	3	K^+^				
	4	Genotype				
BC (g)	**1**	**Fe**^2+^	82.7	57.90	4, 53	3.69
	2	${\text{SO}}_{4}^{2-}$				

*The most significant submodels have been bold by software*.

Table 5 indicates the critical factors (significant *inputs*) for each *output*, together with the Train Set *R*^2^ and ANOVA parameters of the model (calculated *F* ratio, degrees of freedom and *f* critical for α < 0.01). The ANOVA *F* ratio was always greater than the corresponding *f* critical value, indicating good performance and quality of neurofuzzy logic models (Table 5).

Neurofuzzy also succeeded in determining that just 10 out of 19 *inputs* studied (genotype, ${\text{NH}}_{4}^{+}$, ${\text{NO}}_{3}^{-}$, K^+^, ${\text{SO}}_{4}^{2-}$, Cl^−^, Fe^2+^, ${\text{BO}}_{3}^{-}$, Cu^2+^, EDTA^−^) significantly affected the results (Table 5).

Shoot quality score (1 = necrotic, 2 = poor, 3 = moderate, 4 = good) is a subjective measure that encompasses the overall appearance of shoots, including leaf and shoot health as well as multiplication. Neurofuzzy logic split the model for this parameter into four submodels: the interaction ${\text{SO}}_{4}^{2-}$ × Cl^−^, highlighted as the *inputs* with stronger effect (see submodel 2) and the linear independent effects of Fe^2+^, K^+^, and ${\text{NH}}_{4}^{+}$ ions (Table 5).

The “IF-THEN” rules generated by the neurofuzzy models explain in words the effects of the critical *inputs* for each *output*. Table 6 summarizes the combinations of variables to achieve low or high results with the highest degree of membership.

**Table 6:** Rules selection, with membership degree 1.00, generated by neurofuzzy logic showing the best combination of *inputs* to obtain the highest or lowest results for each *output*.

**Rules**		**${\text{NH}}_{4}^{+}$**	**${\text{NO}}_{3}^{-}$**	**K^+^**	**${\text{SO}}_{4}^{2-}$**	**Fe^2+^**	**EDTA^−^**	**Cu^2+^**	**Cl^−^**	**${\text{BO}}_{3}^{-}$**		**SQ**	**PR**	**SL (cm)**	**STN**	**BC (g)**	**Membership degree**
**3**	IF				**Mid**				**Low**		THEN	**High**					**1.00**
**5**					**High**				**Low**			**Low**					**1.00**
6					High				High			High					1.00
7						Low						High					1.00
8						High						Low					1.00
10				High								Low					1.00
11		Low										Low					1.00
13		High										Low					1.00
18	IF			Low	Low		High				THEN		Low				1.00
**19**				**Low**	**High**		**High**						**High**				**1.00**
20				High	Low		High						Low				1.00
**21**				**High**	**High**		**High**						**Low**				**1.00**
24						Mid				Low			High				1.00
28	IF		Low			Low		Low			THEN			Low			1.00
29			Mid			Low		Low						Low			1.00
31			Low			Low		Mid						Low			1.00
32			Mid			Low		Mid						High			1.00
**33**			**High**			**Low**		**Mid**						**High**			**1.00**
34			Low			Low		High						Low			1.00
37			Low			High		Low						Low			1.00
40			Low			High		Mid						Low			1.00
41			Mid			High		Mid						Low			1.00
**42**			**High**			**High**		**Mid**						**Low**			**1.00**
44			Mid			High		High						Low			1.00
45			High			High		High						High			1.00
47				Mid					Low					Low			1.00
**54**	IF						**Low**				THEN				**Low**		**1.00**
**55**							**High**								**High**		**1.00**
56					Low										High		1.00
57					Mid										Low		1.00
58					High										Low		1.00
59				Low											Low		1.00
**64**	IF					**High**					**THEN**					**Low**	**1.00**
65					Low											Low	1.00

*The inputs with stronger effects on each output have been highlighted by software*.

As it can be easily deduced from the rules, the highest shoot quality is achieved with a combination of Mid ${\text{SO}}_{4}^{-2}$ and Low Cl^−^ and Low Fe^2+^ concentration (rules 3 and 7 from Table 6). Negative effects of both K^+^ and ${\text{NH}}_{4}^{+}$ high levels are also deduced (rules 10 and 13 from Table 6). The meaning of Low, Mid or High for the different *inputs* can be found in Figures S1 A1–A4 of Supplementary Materials.

**Figure 2:**
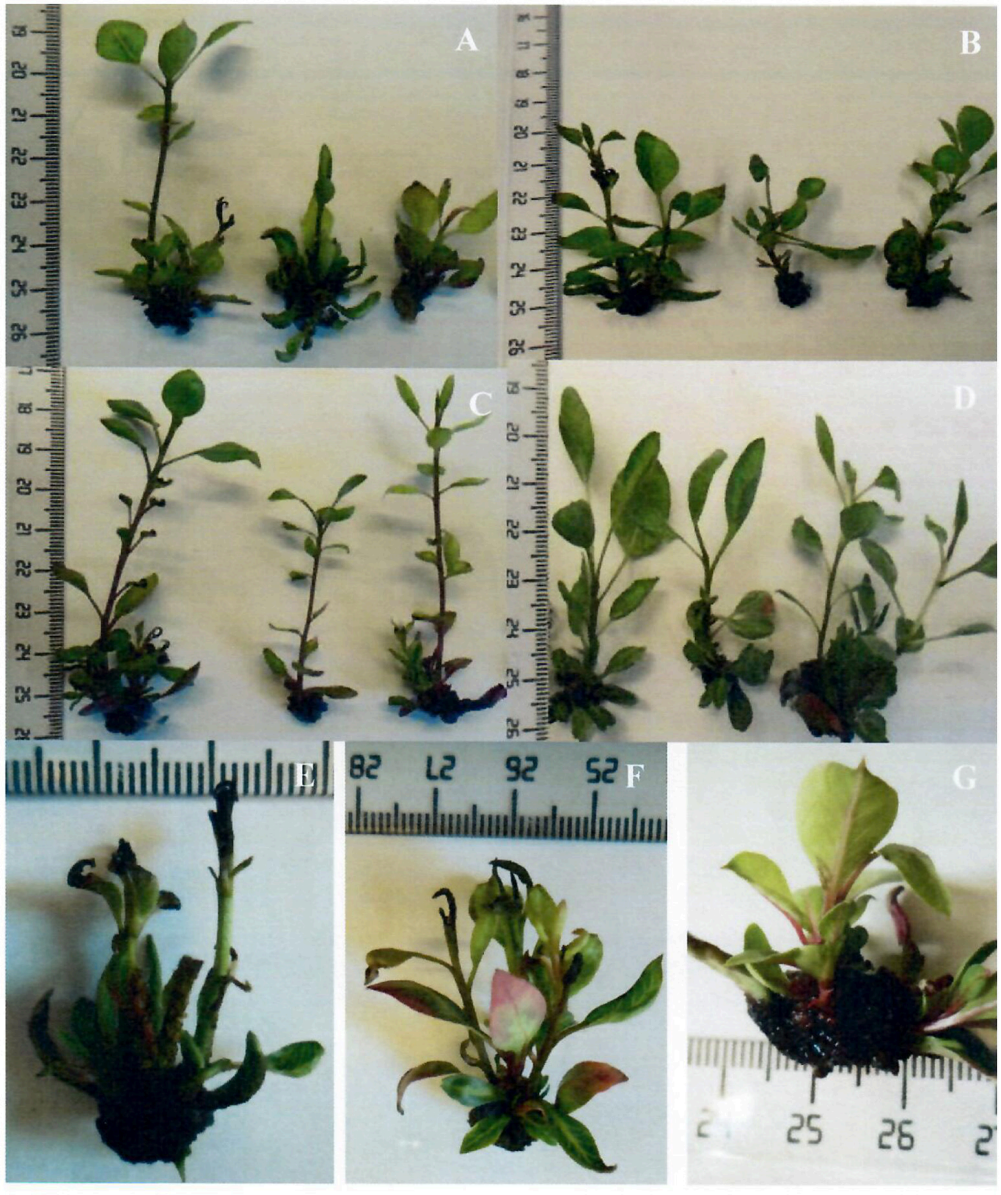
Response of pistachio rootstocks to mineral nutrients “Ghazvini” rootstock on **(A)** MS (control), **(B)** Treatment #24; “UCB1” rootstock on **(C)** MS (control), and **(D)** Treatment #24. The occurrence of some physiological disorders including **(E)** shoot-tip necrosis, leaf necrosis and hooked leaves in “Ghazvini,” **(F)** shoot-tip necrosis in “UCB1”and **(G)** basal callus in “UCB1”.

It is noteworthy that the shoots of both genotypes grown with treatment #24 (Table 4), have a higher quality than those grown with MS (used as control), which agrees with the results of the model (Figures 2A–D).

The new regenerated shoots (PR) variability is explained by two submodels: the complex interaction of K^+^, EDTA^−^ and ${\text{SO}}_{4}^{2-}$ (strongest effect) together with the interaction between Fe^2+^ and ${\text{BO}}_{3}^{-}$ as submodels 1 and 2, respectively (Table 5).

From the corresponding rules (Table 6), two critical conclusions would be extracted: (i) the convenience of using Low EDTA^−^ concentrations (Low < 0.3 mM; Figure S1-B1) for obtaining High PR values, regardless the amounts of either K^+^ or ${\text{SO}}_{4}^{2-}$ (rules 14–17; Table S2); and (ii) the dominant role of K^+^ on the PR parameter when EDTA^−^ and ${\text{SO}}_{4}^{2-}$ are applied at High concentration, producing a set of respective greatest and lowest PR when K^+^ at Low and High amounts (Figure S1-B1) are used (rules 19 and 21; Table 6). Submodel 2 pinpoints the negative influence of ${\text{BO}}_{3}^{-}$ in the presence of Fe^2+^ at either Low or Mid concentrations, giving the highest PR when low amount of ${\text{BO}}_{3}^{-}$ (Figures S1-B2,3) is added to the culture medium (membership degree1.00, rule 24; Table 6).

Neurofuzzy logic explained the shoot length (SL) variability by building three submodels: the complex combination of Fe^2+^, Cu^2+^ and ${\text{NO}}_{3}^{-}$ (strongest effect), the interaction between K^+^ and Cl^−^ ion concentrations and the genotype (submodels 1-3; Table 5).

The analysis of “IF-THEN” rules (Table 6) for this parameter reveals that, if the culture media supplemented with High concentration of ${\text{NO}}_{3}^{-}$ and Mid concentration of Cu^2+^ (Figure S1-C1), the inclusion of Fe^2+^ ion at Low or High concentrations (Figure S1-C2) leads to obtaining the longest or shortest shoots, respectively (rules 33 and 42, Table 6).

According to these rules, the pistachio shoots grown on MS medium (Control), which incorporates Low concentrations of Fe^2+^, medium concentration of Cu^2+^ and high concentration of ${\text{NO}}_{3}^{-}$, has the highest lengths for both genotypes with PR indexes of 2.36 ± 0.70 cm and 1.60 ± 0.14 cm for “UCB1” and “Ghazvini” respectively (Table 4 and Figure 2).

The potassium and chloride ions also affect the growth of the shoots, obtaining the longest shoots when the medium contains Low K^+^ content (<5 mM) together with High Cl^−^ content (>10 mM) (Figures S1-C3, C4) even though with membership 0.71 (rule 49; Table S2).

Lastly, the model also shows differences between genotypes for the shoot length parameter, although high values are obtained for both rootstocks (rules 52-53; Table S2).

In this study, different types of physiological abnormalities such as STN, hyperhydricity and BC during the multiplication of pistachio shoots of both genotypes were observed. Among them, the STN formation and BC were scored and successfully modeled with the neurofuzzy logic tool (Table 5).

Shoot-tip necrosis variability is explained by the linear effect of four *inputs*: EDTA^−^, ${\text{SO}}_{4}^{2-}$, K^+^ and genotype (Table 5 and Figures 2E–F). While the use of EDTA^−^ or K^+^ at Low concentration (Figures S1-D1,3) has deteriorative effect on STN (rules 54 and 59; Table 6), the inclusion of ${\text{SO}}_{4}^{2-}$ has positive effect. The STN is reduced when culture media includes Mid to High amounts of ${\text{SO}}_{4}^{2-}$ (rules 56-58; Table 6 and Figure S1-D2). Finally, results also indicate that the severity of STN in pistachio is significantly dependent on genotype, being its occurrence lower in “UCB1” than in “Ghazvini” (rules 61–62; Table S2).

Callus formation produced at the cut-edge of shoots was found to be dependent of Fe^2+^ and ${\text{SO}}_{4}^{2-}$ ion concentration (Table 5 and Figure 2G). The lowest BC is produced when Fe^2+^ concentration is High, over 0.3 mM (Figure S1-E1) (rules 63-64; Tables 6 and S2). However, it must be noted that although obtaining lowest BC may seems to be best for researchers, in fact the use of Fe^2+^ at excessive concentrations suppress dramatically shoots growth and development (Data not shown). Regarding the role of ${\text{SO}}_{4}^{2-}$ on BC, it is necessary to consider a Low concentration of this ion for preventing BC formation (rules 65–67; Tables 6 and S2, Figure S1-E2).

## Discussion

The ingredients of culture medium, including mineral nutrients, significantly affect pistachio shoots growth (Akdemir et al., 2012; Kilinç et al., 2015; Marín et al., 2017). Recently, a new medium for pistachio was developed using artificial intelligence tools, even with a poorly sampled experimental design (Nezami-Alanagh et al., 2017). Despite of its good results, the study also pointed out the necessity of improving the adjustment in the concentrations of macro- and micro-elements of standard culture media. In this regard, for example, the decrease of KNO_3_ to 558 mg L^−1^ (almost one-fourth) and the increase of MgSO_4_ .7H_2_O up to 468 mg L^−1^ (1.2-fold), CuSO_4_.5H_2_O to 0.11 mg L^−1^ (up to 4.5-fold), FeSO_4_.7H_2_O up to 31.2 mg L^−1^ (1.11-fold) compared to MS salt levels has led to positive effects on the parameters studied (Nezami-Alanagh et al., 2017).

In the present study, we have used a IV-optimal design space using DOE software through dividing macro- and micro-elements of MS medium into five independent factors and assigning various levels for each (Tables 1, 2) with two purposes: (i) to establish a well sampled design space and (ii) to reduce the number of treatments to be assayed from 3125 to just 29 combinations based on MS levels. The use of neurofuzzy logic as an advanced modeling tool (Gago et al., 2010a,b,c,d, 2014; Gallego et al., 2011; Nezami-Alanagh et al., 2014; Ayuso et al., 2017) has enabled determining the key factors affecting the parameters studied. In fact, the efficiency of neurofuzzy logic can be summarized in: (i) constructing statistical significant mathematical models characterized by high coefficients of determination (> 70%) indicating high predictability of the algorithm, since higher the *R*^2^ value obtained, the better the predictability of the trained model (Shao et al., 2006), and (ii) development of submodels defined by a set of vague linguistic tags, expressed as “IF-THEN” rules, that allow the understanding of the complex nonlinear relationships between *inputs* and *outputs* in an easy way.

Shoot quality is a complex parameter that includes the evaluation of several macroscopic observations such as the appearance of shoots health together with absence of physiological disorders (Hand et al., 2014; Niedz et al., 2014). Neurofuzzy logic models have managed to discover the interaction or independent effects of ${\text{SO}}_{4}^{2-}$, Cl^−^, Fe^2+^, K^+^ and ${\text{NH}}_{4}^{+}$ on SQ in pistachio rootstocks with dominant role of ${\text{SO}}_{4}^{2-}$ × Cl^−^ (rules 1–13, Tables 6 and S2). In agreement with our findings, the significant effects of different salts on SQ of different plant species as ZnSO_4_.7H_2_O and Fe/EDTA in *Citrus sinensis* cv. “Valencia” (Niedz and Evens, 2007); *Gerbera hybrida* cv. “Pasadena” (Niedz et al., 2014); CaCl_2_.2H_2_O, MgSO_4_.7H_2_O and KH_2_PO_4_ in *Pyrus sp*. (Wada et al., 2013) and *Rubus idaeus* L. (Poothong and Reed, 2014, 2015); KH_2_PO_4_, K_2_SO_4_ and NH_4_NO_3_ in *Corylus avellana* L. (Akin et al., 2017b)_;_ KH_2_PO_4_ and MgSO_4_.7H_2_O in *Prunus armeniaca* Lam (Kovalchuk et al., 2017) *in vitro* culture have been reported. The use of salts as factors, in all of these studies, implies a problem of ion confounding, being difficult to identify exactly corresponding ion(s) impacting the parameter (Niedz and Evens, 2006). In agreement with our results, Akin et al. (2017a) using CHAID algorithm, found that hazelnut quality was affected by K^+^, ${\text{NO}}_{3}^{-}$, ${\text{NH}}_{4}^{+}$ and genotype, showing that the best SQ for “Barcelona,” “Jefferson,” and “Wepster” was obtained when the culture media was supplemented with K^+^ ≤ 46 mM, ${\text{NO}}_{3}^{-}$ ≤ 88 mM, and ${\text{NH}}_{4}^{+}$ ≤ 20 mM.

Although usually the interaction of ${\text{NH}}_{4}^{+}$ × ${\text{NO}}_{3}^{-}$ × K^+^ has been considered as the central attentions over *in vitro* studies because of their dominant ions in most tissue culture media formulations (Niedz et al., 2014), in present study the implementation of neurofuzzy logic strongly exploited the existence of another kind of relationship between ions (K^+^ × EDTA^−^ × ${\text{SO}}_{4}^{2-}$), with critical influence of K^+^ on PR; so that the integration of K^+^ at Low and High concentrations should lead to achieve a set of greatest and lowest PR, respectively. The beneficial effect of K^+^ at Low amount is in agreement accordance with our earlier study during micropropagation of *Prunus sp*. where K^+^ was integrated in culture media up to 3.19 mM (Nezami-Alanagh et al., 2014), whereas in MS is at 20.05 mM (Table 5). Neurofuzzy logic models also pinpointed that explants cultured on media with Fe^2+^ / EDTA^−^ at Low to Mid concentration, exhibited high PR. Niedz et al. (2014) reported 0.1 mM Fe^2+^ / EDTA^−^ as optimal concentration for micropropagation of *Gerbera hybrida*. Whilst, the use of Fe^2+^ / EDTA^−^ only at 0.23 mM was recommended by Garrison et al. (2013) during micropropagation of hybrid hazelnut. In agreement with those results, here the explants treated with either low or too high amounts of these ions were failure to yield shoots suitable for subculture. This suggests that Fe^2+^ and EDTA^−^ of MS (0.1 mM) can also be used in pistachio micropropagation.

The positive role of ${\text{NO}}_{3}^{-}$ on SL increment, solely or in combination with other ions, has also been reported previously. Wada et al. (2015) using response surface modeling found that to attain longest shoots in diverse pear species it is necessary to integrate High ${\text{NO}}_{3}^{-}$ [20–60 mM] in addition to a proper range of ${\text{NH}}_{4}^{+}$: K^+^ ratios to culture medium. In the previous study using neurofuzzy logic we found that ${\text{NO}}_{3}^{-}$ at 17.5–20 m M led to produce longest shoots in *P. vera* cv. ‘Ghazvini’ (Nezami-Alanagh et al., 2017). Interestingly, the experimental design used in the current study allowed to have global viewpoint related to role of this ion on the parameter studied. So that, based on neurofuzzy logic models it was revealed that combination of high ${\text{NO}}_{3}^{-}$ with low Fe^2+^ and Mid Cu^2+^ being necessary to achieve long shoots (Rule 33; Table 6). Niedz et al. (2014) determined the optimal amount of some chemicals including Fe^2+^ / EDTA^−^ and CuSO_4_.5H_2_O in a single-factor design, followed by illustrating 0.1 and 0-0.1 mM as the optimum concentration for obtaining longest shoots in *Gerbera hybrid* together with the effects of deterioration of these metals on the parameters at higher concentration which agrees with our findings. Moreover, the interaction K^+^ × Cl^−^ was also determined as the next significant factors on SL parameter, promoting longest shoots IF Low K^+^ plus High Cl^−^ is included in the culture media. It is interesting to note that neurofuzzy logic generated useful information about the importance of ${\text{NO}}_{3}^{-}$, Cu^2+^ and Fe^2+^, suggesting that the first should be used at lower concentration (20 mM) compared with that use in MS (39 mM).

In this study some physiological disorders such as STN and BC are described. STN has been considered as one of the main drawbacks in micropropagation of different species including *Pistacia* (Onay, 2003; Bairu et al., 2009a; Bariu et al., 2009b; Chiruvella et al., 2011; Akdemir et al., 2014) and has been attributed to certain mineral nutrients deficiencies such as calcium and/or boron (Barghchi and Alderson, 1996; Akdemir et al., 2012). Neurofuzzy logic has pointed out the significant influence of other factors on this type of abnormality. EDTA^−^ has been highlighted by the model as the factor with the strongest effect on STN, since at High concentration promotes the highest STN (rule 55; Table 6). Probable reasons explaining this effect could be the toxicity effects of EDTA^−^ when using at excessive amounts or chelating with other metals causing mineral deficiency in shoots (George et al., 2008). Furthermore, production of formaldehyde as a result of the EDTA^−^ oxidization mediated by the fluorescence lamps within the growth chamber, which can also became accumulated until inhibitory levels (Hangarter and Stasinopoulos, 1991). Therefore, Low EDTA^−^ (0.1 mM) should be used for healthy pistachio micropropagation.

Reed et al. (2013) using RSM methodology reported that low levels of some MS ingredients such as KH_2_PO_4_, MgSO_4_.7H_2_O, CaCl_2_.2H_2_O, NH_4_NO_3_ and KNO_3_ contributed to promote necrosis in some species of *Pyurus* including *P. ussuriensis* ‘Hang Pa Li’. Again, the problem of ion confounding prevents the establishment of causality between the specific ions and the physiological abnormality. Our results clearly determined that low concentrations of K^+^ and Mid-High level of ${\text{SO}}_{4}^{2-}$ inhibit the STN symptoms (rules 56-60; Tables 6 and S2). Finally, neurofuzzy logic pointed out additionally, the differences between genotypes with regard STN parameter, being the occurrence of the symptoms lower in “UCB1” than in “Ghazvini” (rules 61–62; Table S2).

Callus formation at the base of shoots is also considered as one of common *in vitro* physiological disorders in diverse woody plants species, which is affected mainly by chemical composition of culture medium. For instance, the integration of some mineral ingredients at a certain concentrations such as KH_2_PO_4_, MgSO_4_ and CaCl_2_ in some *Prunus* cultivars (Reed et al., 2013), MgSO_4_·7H_2_O in *Prunus armeniaca* Lam., (Kovalchuk et al., 2017), or ${\text{NO}}_{3}^{-}$ ion in Robus germplasms (Poothong and Reed, 2016) caused basal callus formation of *in vitro* shoots. In a recent study using CHAID analysis has been reported the significant effects of ${\text{NH}}_{4}^{+}$, followed by genotype and ${\text{SO}}_{4}^{2-}$ on callus formation in hazelnut shoots (Akin et al., 2017a). Neurofuzzy logic has pinpointed the independent significant effect of Fe^2+^ and ${\text{SO}}_{4}^{2-}$ as the two most important factors on this parameter, predicting the lowest BC in pistachio rootstocks when these components are used at High and Low concentrations, respectively (rules 63–67; Tables 6 and S2). These results suggest that ${\text{SO}}_{4}^{2-}$ at lower concentration than in MS (1.173 mM) is better to reduce STN and BC in pistachio.

## Conclusions

From this study two main conclusions can be drawn. First, pistachio growth parameters SQ, PR, SL, STN and BC can be improved by using medium which include the next ions concentration: K^+^ (<10 mM); Fe^2+^ and EDTA^−^ (0.1 mM); ${\text{BO}}_{3}^{-}$ (<0.2 mM); Cu^2+^ (≈0.0002 mM); ${\text{NO}}_{3}^{-}$ (<20 mM) and ${\text{SO}}_{4}^{2-}$ (≈4 mM). This combination, which is in accordance with our previous studies (Nezami-Alanagh et al., 2017), allows to increase the healthy micropropagation of pistachio.

Second, the acquisition of knowledge about the performance of complex systems, such as *in vitro* culture, can be greatly increased by combining the use of DOE and computer-based mathematical models generated by artificial intelligence. While DOE lets to reduce the number of treatments in complex factorial designs, but ensuring a good sampled design space, neurofuzzy logic models facilitate the analysis of large databases and the establishment of the critical factors for all parameters studied, simultaneously.

## Author contributions

EN-A performed the experiments. G-AG contributed with reagents materials. EN-A, G-AG, and PG conceived and designed the experiments. ML and PG contributed DOE/modeling/analysis tools. All authors contributed to writing of the manuscript.

### Conflict of interest statement

The authors declare that the research was conducted in the absence of any commercial or financial relationships that could be construed as a potential conflict of interest.

## Supplementary material

Figure S1 Graphical representation of the fuzzyfication process developed by neurofuzzy logic per each parameter **(A1–A4)** shoot quality, **(B1–B3)** Proliferation rate, **(C1–C4)** shoot length (in cm), **(D1–D3)** shoot-tip necrosis and **(E1,2)** basal callus (in gram) and their domains (Low, Mid, High, ….). Neurofuzzy divided the input range (*x* axis) of a variable between several domains to which a symbolic name is attached. In this sense, two (Low and High) and three (Low, Mid and High) were established for the significant submodels developed by neurofuzzy logic for each parameter (see Table 5). The *y* axis represents the membership function ranging from 0 to 1: the closer the membership function is to 1, the closer the value is to truly Low or truly High (if two domains were established, as for Cl^−^ in **A2**). This process facilitate the interaction between the membership function (0 and 1) and the linguistic terms (Low, Mid, High) making the terms meaningful to a computer. After the modeling the knowledge extracted is presented by “IF-THEN” rules. As example, if we refer in Rule 3 Mid ${\text{SO}}_{4}^{2-}$ concentration having a look to panel **A1**, this means around 3 mM with a membership degree of 1.00, whereas 4 mM can be either considered Mid (membership 0.6) or Low (membership 0.3). In other words, ${\text{SO}}_{4}^{2-}$ at 3 mM is pondered by the model as Mid concentration rather than low and never High (membership 0).

## References

[B1] Abousalim A. Mantell S. H. (1992). Micrografting of pistachio (*Pistacia vera* L. cv. Mateur). Plant Cell Tissue Org. Cult. 29, 231–234.

[B2] Abousalim A. Mantell S. H. (1994). A practical method for alleviating shoot-tip necrosis symtoms in *in vitro* shoot cultures of *Pistacia vera* cv. Mateur. J. Hortic. Sci. 69, 357–365.

[B3] Akdemir H. Süzerer V. Onay A. Tilkat E. Ersali Y. Çiftçi Y. O. (2014). Micropropagation of the pistachio and its rootstocks by temporary immersion system. Plant Cell Tissue Org. Cult. 117, 65–76. 10.1007/s11240-013-0421-0

[B4] Akdemir H. Yildirim H. Tilkat E. Onay A. Özden Çiftçi Y. (2012). Prevention of shoot tip necrosis responses in *in vitro*-proliferated mature pistachio plantlets. in Vitro Biology Meeting of the SIVB. Seattle, WA, P-2003.

[B5] Akin M. Eyduran E. Niedz R. P. Reed B. M. (2017a). Developing hazelnut tissue culture medium free of ion confounding. Plant Cell Tissue Org. Cult. 130, 483–494. 10.1007/s11240-017-1238-z

[B6] Akin M. Eyduran E. Reed B. M. (2017b). Use of RSM and CHAID data mining algorithm for predicting mineral nutrition of hazelnut. Plant Cell Tissue Org. Cult. 128, 303–316. 10.1007/s11240-016-1110-6

[B7] Ayuso M. Ramil-Rego P. Landin M. Gallego P. P. Barreal M. E. (2017). Computer-assisted recovery of threatened plants: keys for breaking seed dormancy of eryngium viviparum. Front. Plant Sci. 8:2092. 10.3389/fpls.2017.02092 29312370 PMC5732921

[B8] Bairu M. W. Jain N. Stirk W. A. DoleŽal K. Van Staden J. (2009a). Solving the problem of shoot-tip necrosis in *Harpagophytum* procumbens by changing the cytokinin types, calcium and boron concentrations in the medium. S. afr. J. Bot. 75, 122–127. 10.1016/j.sajb.2008.08.006

[B9] Barghchi M. Alderson P. G. (1983a). *In vitro* propagation of *Pistacia* species. Acta Hortic. 131, 49–60.

[B10] Barghchi M. Alderson P. G. (1983b). In vitro propagation of *Pistacia vera L* from seedling tissues. J. Hortic. Sci. 58, 435–445.

[B11] Barghchi M. Alderson P. G. (1985). *In vitro* propagation of *Pistacia vera L*. and the commercial cultivars Ohadi and Kalleghochi. J. Hortic. Sci. 60, 423–430.

[B12] Barghchi M. Alderson P. G. (1996). The control of shoot tip necrosis in *Pistacia vera L*. in vitro. J. Plant Growth Regul. 20, 31–35.

[B13] Bariu W. M. Stirk W. A. Van Staden J. (2009b). Factors contributing to *in vitro* shoot-tip necrosis and their physiological interactions. Plant Cell Tissue Org. Cult. 98, 239–248. 10.1007/s11240-009-9560-8

[B14] Benmahioul B. Dorion N. Kaid-Harche M. Daguin F. (2012). Micropropagation and *ex vitro* rooting of pistachio (*Pistacia vera* L.). Plant Cell Tissue Org. Cult. 108, 353–358. 10.1007/s11240-011-0040-6

[B15] Benmahioul B. Kaid-Harche M. Dorion N. Daguin F. (2009). *In vitro* embryo germination and proliferation of pistachio (*Pistacia ver*a L.). Sci. Hortic. 122, 479–483. 10.1016/j.scienta.2009.05.029

[B16] Chiruvella K. K. Mohammed A. Dampuri G. Ghanta R. G. (2011). *In vitro* shoot regeneration and control of shoot tip necrosis in tissue cultures of soymida febrifuga (Roxb.) A. Juss. Plant Tissue Cult. Biotech. 21, 11–25. 10.3329/ptcb.v21i1.9559

[B17] Colbourn E. A. Rowe R. C. (2005). Neural computing and pharmaceutical formulation, in Encyclopaedia of Pharmaceutical Technology, eds Swarbrick J. Boylan J. (New York, NY: Marcel Dekker), 145–157.

[B18] Colbourn E. A. Rowe R. C. (2009). Novel approaches to neural and evolutionary computing in pharmaceutical formulation: challenges and new possibilities. Fut. Med. Chem. 1, 713–726. 10.4155/fmc.09.57 21426034

[B19] Design-Expert (2010). Design-Expert. Minneapolis, MN: Stat-Ease, Inc.

[B20] Dolcet-Sanjuan R. Claveria E. (1995). Improved shoot-tip micropropagation of *Pistacia vera* L. and the beneficial effects of methyl jasmonate. J. Am. Soc. Hort. Sci. 120, 938–942.

[B21] Driver J. A. Kuniyuki A. H. (1984). *In vitro* propagation of Paradox walnut rootstock. HortScience 19, 507–509.

[B22] Gago J. Landín M. Gallego P. P. (2010a). A neurofuzzy logic approach for modeling plant processes: a practical case of *in vitro* direct rooting and acclimatization of *Vitis vinifera* L. Plant Sci. 179, 241–249. 10.1016/j.plantsci.2010.05.009

[B23] Gago J. Landín M. Gallego P. P. (2010b). Artificial neural networks modeling the *in vitro* rhizogenesis and acclimatization of *Vitis vinifera* L. J. Plant Physiol. 167, 1226–1231. 10.1016/j.jplph.2010.04.008 20542352

[B24] Gago J. Landín M. Gallego P. P. (2010c). Strengths of artificial neural networks in modelling complex plant processes. Plant Signal. Behav. 5, 743–745. 10.4161/psb.5.6.11702 20421726 PMC3001577

[B25] Gago J. Martínez-Núñez L. Landín M. Flexas J. Gallego P. P. (2014). Modeling the effects of light and sucrose on *in vitro* propagated plants: a multiscale system analysis using artificial intelligence technology. PLoS ONE 9:e85989. 10.1371/journal.pone.0085989 24465829 PMC3896442

[B26] Gago J. Martínez-Núñez L. Landín M. Gallego P. P. (2010d). Artificial neural networks as an alternative to the traditional statistical methodology in plant research. J. Plant Physiol. 167, 23–27. 10.1016/j.jplph.2009.07.007 19716625

[B27] Gago J. Pérez-Tornero O. Landín M. Burgos L. Gallego P. P. (2011). Improving knowledge of plant tissue culture and media formulation by neurofuzzy logic: a practical case of data mining using apricot databases. J. Plant Physiol. 168, 1858–1865. 10.1016/j.jplph.2011.04.008 21676490

[B28] Gallego P. P. Gago J. Landín M. (2011). Artificial neural networks technology to model and predict plant biology process, in Artificial Neural Networks-Methodological Advances and Biomedical Applications, ed Suzuki K. (Rijeka: Intech Open Access Publisher), 197–216.

[B29] Garrison W. Dale A. Saxena P. K. (2013). Improved shoot multiplication and development in hybrid hazelnut nodal cultures by ethylenediamine di-2-hydroxy-phenylacetic acid (Fe-EDDHA). Can. J. Plant Sci. 93, 511–521. 10.4141/cjps2012-218

[B30] George E. F. Hall M. A. De Klerk G.-J. (eds.). (2008). The Components of Plant Tissue Culture Media I: Macro- and Micro-Nutrients, in Plant Propagation by Tissue Culture (Dordrecht: Springer), 65–113. 10.1007/978-1-4020-5005-3_3

[B31] Gonzalez A. Frutos D. (1990). *In vitro* culture of *Pistacia vera* L. embryos and aged trees explants, in Plant Aging: Basic and Applied Approaches, eds Rodriguez R. Sanchez Tames R. Durzan D. J. (New York, NY; London: Plenum Press (in cooperation with NATO Scientific Affairs Division)), 335–338.

[B32] Hand C. Maki S. Reed B. M. (2014). Modeling optimal mineral nutrition for hazelnut micropropagation. Plant Cell Tissue Org. Cult. 119, 411–425. 10.1007/s11240-014-0544-y

[B33] Hangarter R. P. Stasinopoulos T. C. (1991). Effect of Fe-catalyzed photooxidation of EDTA on root growth in plant culture media. Plant Physiol. 96, 843–847. 16668263 10.1104/pp.96.3.843 PMC1080853

[B34] Karimi S. Rahemi M. Maftoun M. Tavallali E. Tavallali V. (2009). Effects of long-term salinity on growth and performance of two pistachio (*Pistacia* L.) rootstocks. Aust. J. Basic Appl. Sci. 3, 1630–1639.

[B35] Kilinç F. M. Süzerer V. Çiftçi Y. Ö. Onay A. Yildirim H. Uncuoglu A. A. (2015). Clonal micropropagation of *Pistacia lentiscus* L. and assessment of genetic stability using IRAP markers. J. Plant Growth Regul. 75, 75–88. 10.1007/s10725-014-9933-9

[B36] Kovalchuk I. Y. Mukhitdinova Z. Turdiyev T. Madiyeva G. Akin M. Eyduran E. (2017). Modeling some mineral nutrient requirements for micropropagated wild apricot shoot cultures. Plant Cell Tissue Org. Cult. 129, 325–335. 10.1007/s11240-017-1180-0

[B37] Lloyd G. McCown B. (1980). Commercially-feasible micropropagation of mountain laurel, *Kalmia latifolia*, by use of shoot-tip culture. Proc. Int. Plant Prop. Soc. 30, 421–427.

[B38] Marín J. A. García E. Lorente P. Arbeloa A. Andreu P. (2017). Propagation of pistachio applying *in vitro* culture techniques. Acta Hortic. 1155, 321–326. 10.17660/ActaHortic.2017.1155.46

[B39] Mederos-Molina S. Trujillo M. I. (1999). Elimination of browning exudate and *in vitro* development of shoots in *Pistacia vera* L. cv. mateur and *Pistacia atlantica* desf. culture. Acta Soc. Bot. Pol. 68, 21–24.

[B40] Murashige T. Skoog F. (1962). A revised medium for rapid growth and bio assays with tobacco tissue cultures. Physiol. Plant. 15, 473–497.

[B41] Nezami-Alanagh E. Garoosi G. A. Haddad R. Maleki S. Landin M. Gallego P. P. (2014). Design of tissue culture media for efficient *Prunus* rootstock micropropagation using artificial intelligence models. Plant Cell Tissue Org. Cult. 117, 349–359. 10.1007/s11240-014-0444-1

[B42] Nezami-Alanagh E. Garoosi G. A. Maleki S. Landín M. Gallego P. P. (2017). Predicting optimal *in vitro* culture medium for *Pistacia vera* micropropagation using neural networks models. Plant Cell Tissue Org. Cult. 129, 19–33. 10.1007/s11240-016-1152-9

[B43] Niedz R. P. Evens T. J. (2006). A solution to the problem of ion confounding in experimental biology. Nat. Methods 3:417. 10.1038/nmeth0606-417 16721374

[B44] Niedz R. P. Evens T. J. (2007). Regulating plant tissue growth by mineral nutrition. In Vitro Cell Dev. Biol. Plant 43, 370–381. 10.1007/s11627-007-9062-5

[B45] Niedz R. P. Evens T. J. (2016). Design of experiments (DOE)—history, concepts, and relevance to *in vitro* culture. In Vitro Cell Dev. Biol. Plant 52, 547–562. 10.1007/s11627-016-9786-1

[B46] Niedz R. P. Hyndman S. E. Evens T. J. Weathersbee A. A. (2014). Mineral nutrition and *in vitro* growth of *Gerbera hybrida* (Asteraceae). In Vitro Cell Dev. Biol. Plant 50, 458–470. 10.1007/s11627-014-9620-6

[B47] Onay A. (1996). In vitro Organogenesis and Embryogenesis of Pistachio, Pistacia vera L. Ph.D. thesis, University of Edinburgh, UK.

[B48] Onay A. (2000). Micropropagation of Pistachio from mature trees. Plant Cell Tissue Org. Cult. 60, 159–163. 10.1023/A:1006423914951

[B49] Onay A. (2003). Micropropagation of pistachio, in Micropropagation of Woody Trees and Fruits, eds Jain S. M. Ishii K. (Dordrecht: Kluwer Academic Publishers), 565–588.

[B50] Parfitt D. E. Almehdi A. A. (1994). Use of high CO_2_ atmosphere and medium modifications for the successful micropropagation of pistachio. Sci. Hortic. 56, 321–329.

[B51] Poothong S. Reed B. M. (2014). Modeling the effects of mineral nutrition for improving growth and development of micropropagated red raspberries. Sci. Hortic. 165, 132–141.

[B52] Poothong S. Reed B. M. (2015). Increased CaCl_2_, MgSO_4_, and KH_2_PO_4_ improve the growth of micropropagated red raspberries. In Vitro Cell Dev. Biol. Plant 51, 648–658. 10.1007/s11627-015-9720-y

[B53] Poothong S. Reed B. M. (2016). Optimizing shoot culture media for Rubus germplasm: the effects of ${\text{NH}}_{4}^{+}$, ${\text{NO}}_{3}^{-}$, and total nitrogen. In Vitro Cell Dev. Biol. Plant 52, 265–275. 10.1007/s11627-016-9750-0

[B54] Reed B. M. DeNoma J. Wada S. Niedz R. (2016). Determining optimum *in vitro* mineral nutrition for diverse pear germplasm using response surface methodology. Acta Hortic. 1113, 79–84. 10.17660/ActaHortic.2016.1113.11

[B55] Reed B. M. Wada S. DeNoma J. Niedz R. P. (2013). Mineral nutrition influences physiological responses of pear *in vitro*. In Vitro Cell Dev. Biol. Plant 49, 699–709. 10.1007/s11627-013-9556-2

[B56] Shao Q. Rowe R. C. York P. (2006). Comparison of neurofuzzy logic and neural networks in modelling experimental data of an immediate release tablet formulation. Eur. J. Pharm. Sci. 28, 394–404. 10.1016/j.ejps.2006.04.007 16781126

[B57] Tilkat E. Işikalan Ç. Onay A. (2005). *In vitro* propagation of khinjuk pistachio (*Pistacia khinjuk* Stocks) through seedling apical shoot tip culture. Propag. Ornam. Plants 5, 124–128.

[B58] Tilkat E. Onay A. Yildirim H. Çetin Ozen H. (2008). Micropropagation of mature male pistachio *Pistacia vera* L. J. Hortic. Sci. Biotechnol. 83, 328–333. 10.1080/14620316.2008.11512387

[B59] Wada S. Niedz R. P. DeNoma J. Reed B. M. (2013). Mesos components (CaCl_2_, MgSO_4_, KH_2_PO_4_) are critical for improving pear micropropagation. In Vitro Cell Dev. Biol. Plant 49, 356–365. 10.1007/s11627-013-9508-x

[B60] Wada S. Niedz R. P. Reed B. M. (2015). Determining nitrate and ammonium requirements for optimal *in vitro* response of diverse pear species. In Vitro Cell Dev. Biol. Plant 51, 19–27. 10.1007/s11627-015-9662-4

[B61] Yang Z. Lüdders P. (1993). *In vitro* propagation of pistachio (*Pistacia vera* L.). Gartenbauwissenschaf. 59, 30–34.

