# Supplementary figures and images for: Combining DOE With Neurofuzzy Logic for Healthy Mineral Nutrition of Pistachio Rootstocks in vitro Culture
Source: Front Plant Sci. 2018 Oct 15;9:1474. doi: 10.3389/fpls.2018.01474 (PMC6196285; doi:10.3389/fpls.2018.01474)

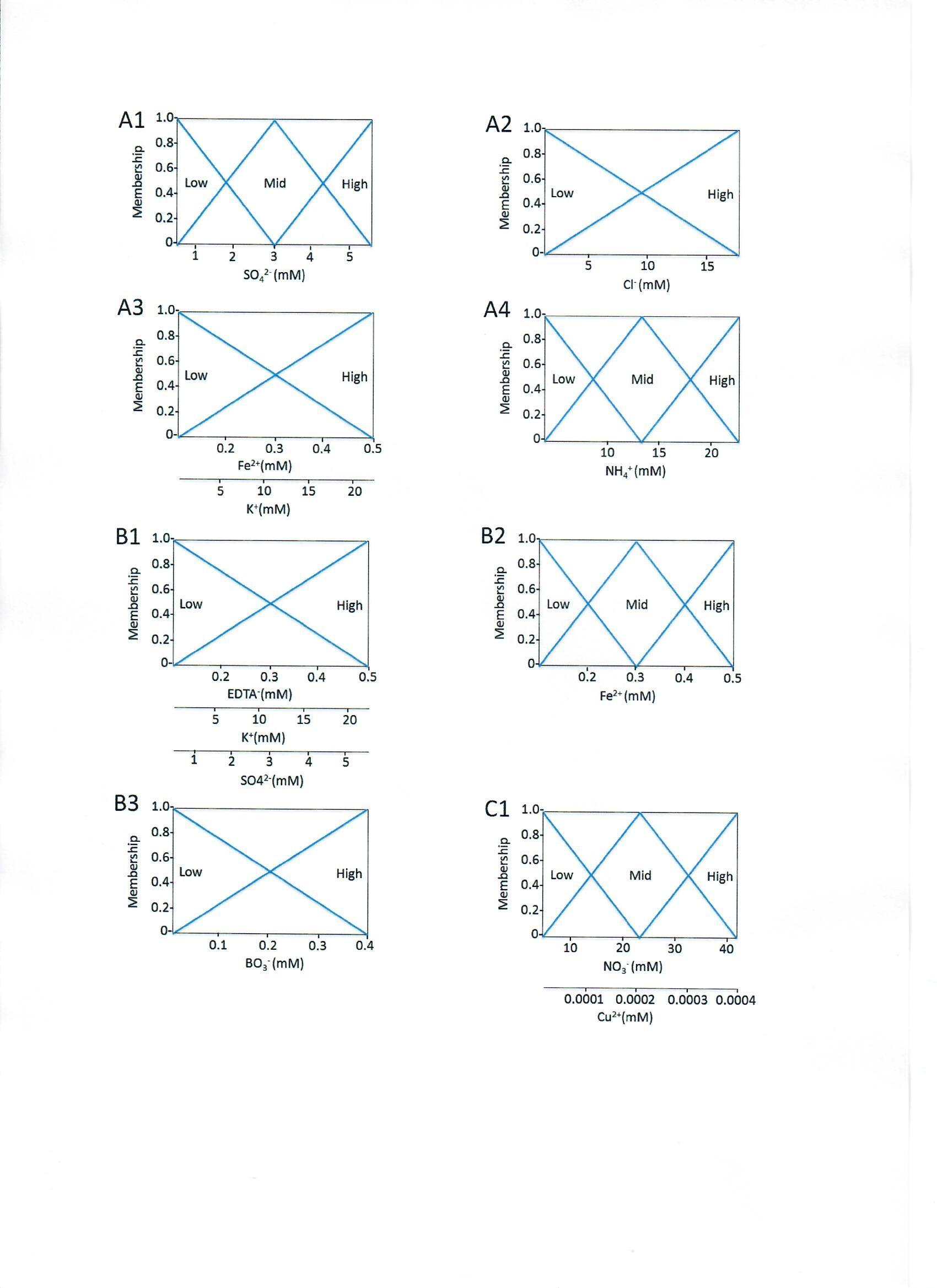

Supplement: Figure S1 — Graphical representation of the fuzzyfication process developed by neurofuzzy logic per each parameter (A1–A4) shoot quality, (B1–B3) Proliferation rate, (C1–C4) shoot length (in cm), (D1–D3) shoot-tip necrosis and (E1,2) basal callus (in gram) and their domains (Low, Mid, High, ….). Neurofuzzy divided the input range (x axis) of a variable between several domains to which a symbolic name is attached. In this sense, two (Low and High) and three (Low, Mid and High) were established for the significant submodels developed by neurofuzzy logic for each parameter (see Table 5). The y axis represents the membership function ranging from 0 to 1: the closer the membership function is to 1, the closer the value is to truly Low or truly High (if two domains were established, as for Cl− in A2). This process facilitate the interaction between the membership function (0 and 1) and the linguistic terms (Low, Mid, High) making the terms meaningful to a computer. After the modeling the knowledge extracted is presented by “IF-THEN” rules. As example, if we refer in Rule 3 Mid SO42- concentration having a look to panel A1, this means around 3 mM with a membership degree of 1.00, whereas 4 mM can be either considered Mid (membership 0.6) or Low (membership 0.3). In other words, SO42- at 3 mM is pondered by the model as Mid concentration rather than low and never High (membership 0). [file Image_1.JPEG]

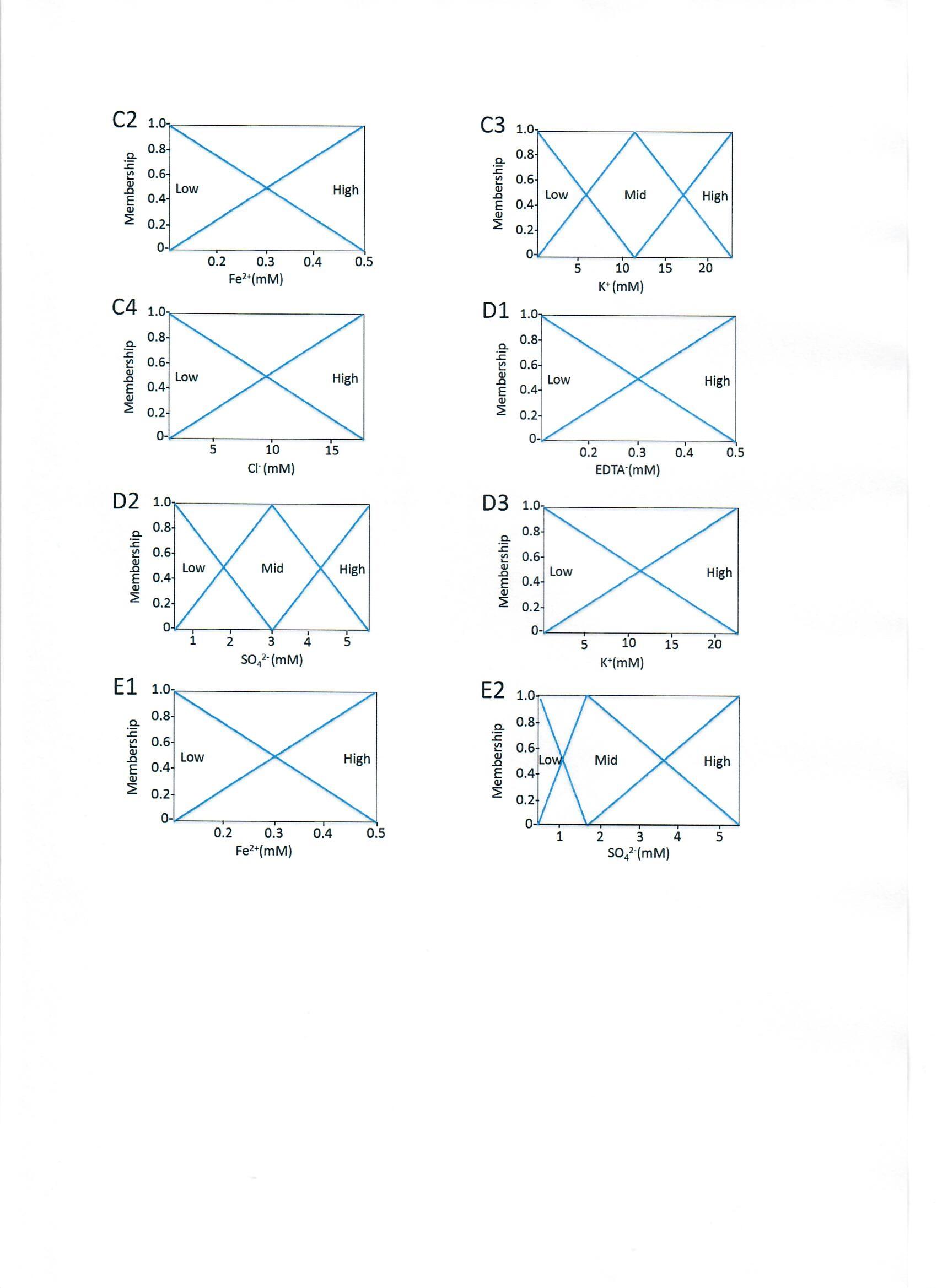

Supplement: Supplementary file 2 [file Image_2.JPEG]
