# Supplementary material for: Combining DOE With Neurofuzzy Logic for Healthy Mineral Nutrition of Pistachio Rootstocks in vitro Culture
Source: Front Plant Sci. 2018 Oct 15;9:1474. doi: 10.3389/fpls.2018.01474 (PMC6196285; doi:10.3389/fpls.2018.01474)
Supplement: Supplementary file 3 [file Table_1.DOC]

**TABLE S1|** Salt mineral composition of the different culture media based on five factor design space to optimize micropropation of *P. vera* rootstocks

| **Trteatment** | **Genotype** | **Salts (mg L^-1^)** | | | | | | | | | | | | | |  | **Growth parameters** | | |  | Physiological disorders | |
| --- | --- | --- | --- | --- | --- | --- | --- | --- | --- | --- | --- | --- | --- | --- | --- | --- | --- | --- | --- | --- | --- | --- |
|  |  | **KNO_3_** | **NH_4_NO_3_** | **CaCl_2_**  **•2H_2_O** | **MgSO_4_**  **•7H_2_O** | **KH_2_PO_4_** | **MnSO_4_**  **•4H_2_O** | **ZnSO_4_**  **•7H_2_O** | **H_3_BO_3_** | **KI** | **CuSO_4_**  **•5H_2_O** | **Na_2_MoO_4_**  **•2H_2_O** | **CoCl_2_**  **•6H_2_O** | **FeSO_4_**  **•7H_2_O** | **Na_2_ •EDTA**  **•7H_2_O** |  | **SQ** | **PR** | **SL (cm)** |  | **STN** | **BC (g)** |
| #1 | UCB1 | 1900 | 330 | 110 | 92.5 | 42.5 | 89.2 | 34.4 | 24.8 | 3.32 | 0.1 | 1 | 0.1 | 65.09 | 87.03 |  | 1.89±0.16 | 2.40±0.40 | 0.74±0.07 |  | 1.88±0.18 | 0.128±0.010 |
| #2 | UCB1 | 0 | 330 | 916.66 | 770.83 | 354.16 | 89.2 | 34.4 | 24.8 | 3.32 | 0.1 | 1 | 0.1 | 27.9 | 37.3 |  | 2.10±0.13 | 2.85±0.28 | 0.62± 004 |  | 1.97±0.27 | 0.194±0.017 |
| #3 | UCB1 | 1900 | 1815 | 916.66 | 770.83 | 354.16 | 2.23 | 0.86 | 0.62 | 0.083 | 0.0025 | 0.025 | 0.0025 | 27.9 | 37.3 |  | 2.73±0.13 | 2.50±0.19 | 0.77±0.05 |  | 1.05±0.05 | 0.225±0.016 |
| #4 | UCB1 | 0 | 1815 | 110 | 92.5 | 42.5 | 31.22 | 12.04 | 8.68 | 1.162 | 0.035 | 0.35 | 0.035 | 102.30 | 136.76 |  | 1.72±0.16 | 1.56±0.29 | 0.81±0.09 |  | 2.61±0.32 | 0.006±0.002 |
| #5 | UCB1 | 0 | 1815 | 1320 | 1110 | 510 | 89.2 | 34.4 | 24.8 | 3.32 | 0.1 | 1 | 0.1 | 27.9 | 37.3 |  | 3.00±0.07 | 3.20±0.38 | 1.35±0.15 |  | 1.05±0.05 | 0.245±0.029 |
| #6 | UCB1 | 950 | 330 | 1320 | 1110 | 510 | 2.23 | 0.86 | 0.62 | 0.083 | 0.0025 | 0.025 | 0.0025 | 139.5 | 186.5 |  | 1.00±0.00 | 0 | 0 |  | 4.00±0.00 | 0 |
| #7 | UCB1 | 0 | 330 | 110 | 92.5 | 42.5 | 2.23 | 0.86 | 0.62 | 0.083 | 0.0025 | 0.025 | 0.0025 | 27.9 | 37.3 |  | 2.65±0.18 | 3.00±0.39 | 0.51±0.06 |  | 1.00±0.00 | 0.059±0.014 |
| #8 | UCB1 | 0 | 1815 | 1320 | 1110 | 510 | 89.2 | 34.4 | 24.8 | 3.32 | 0.1 | 1 | 0.1 | 139.5 | 186.5 |  | 2.00±0.22 | 2.90±0.31 | 0.57±0.08 |  | 2.25±0.35 | 0.074±0.010 |
| #9 | UCB1 | 633.33 | 825 | 110 | 92.5 | 42.5 | 2.23 | 0.86 | 0.62 | 0.083 | 0.0025 | 0.025 | 0.0025 | 139.5 | 186.5 |  | 1.00±0.00 | 0 | 0 |  | 4.00±0.00 | 0 |
| #10 | UCB1 | 0 | 825 | 1320 | 1110 | 510 | 2.23 | 0.86 | 0.62 | 0.083 | 0.0025 | 0.025 | 0.0025 | 65.09 | 87.03 |  | 2.44±0.29 | 3.89±0.35 | 0.45±0.05 |  | 1.55±0.29 | 0.143±0.016 |
| #11 | UCB1 | 1900 | 1815 | 110 | 92.5 | 42.5 | 89.2 | 34.4 | 24.8 | 3.32 | 0.1 | 1 | 0.1 | 27.9 | 37.3 |  | 1.40±0.16 | 2.30±0.47 | 0.62±0.11 |  | 1.60±0.30 | 0.112±0.022 |
| #12 | UCB1 | 0 | 330 | 513.33 | 431.66 | 198.33 | 60.21 | 23.22 | 16.74 | 2.241 | 0.0675 | 0.675 | 0.0675 | 139.5 | 186.5 |  | 2.28±0.27 | 2.44±0.24 | 0.68±0.07 |  | 1.77±0.32 | 0.040±0.006 |
| #13 | UCB1 | 1900 | 1072.5 | 110 | 92.5 | 42.5 | 2.23 | 0.86 | 0.62 | 0.083 | 0.0025 | 0.025 | 0.0025 | 27.9 | 37.3 |  | 1.94±0.05 | 2.89±0.35 | 0.46±0.04 |  | 3.05±0.21 | 0.120±0.008 |
| #14 | UCB1 | 633.33 | 330 | 1320 | 1110 | 510 | 89.2 | 34.4 | 24.8 | 3.32 | 0.1 | 1 | 0.1 | 102.30 | 136.76 |  | 2.39±0.13 | 3.11±0.26 | 0.62±0.07 |  | 1.22±0.22 | 0.184±0.019 |
| #15 | UCB1 | 1900 | 330 | 513.33 | 431.66 | 198.33 | 2.23 | 0.86 | 0.62 | 0.083 | 0.0025 | 0.025 | 0.0025 | 102.30 | 136.76 |  | 1.63±0.21 | 2.05±0.22 | 0.62±0.07 |  | 2.67±0.32 | 0.079±0.005 |
| #16 | UCB1 | 1266.66 | 1815 | 1320 | 1110 | 510 | 2.23 | 0.86 | 0.62 | 0.083 | 0.0025 | 0.025 | 0.0025 | 102.30 | 136.76 |  | 2.19±0.33 | 2.50±0.37 | 0.56±0.08 |  | 1.75±0.41 | 0.106±0.015 |
| #17 | UCB1 | 0 | 1815 | 715 | 601.25 | 276.25 | 2.23 | 0.86 | 0.62 | 0.083 | 0.0025 | 0.025 | 0.0025 | 27.9 | 37.3 |  | 2.78±0.22 | 2.78±0.32 | 0.50±0.05 |  | 1.11±0.11 | 0.249±0.054 |
| #18 | UCB1 | 1900 | 1320 | 1320 | 1110 | 510 | 89.2 | 34.4 | 24.8 | 3.32 | 0.1 | 1 | 0.1 | 65.09 | 87.03 |  | 3.31±0.36 | 2.50±0.42 | 1.23±0.21 |  | 1.56±0.37 | 0.190±0.023 |
| #19 | UCB1 | 1900 | 1320 | 110 | 92.5 | 42.5 | 60.21 | 23.22 | 16.74 | 2.241 | 0.0675 | 0.675 | 0.0675 | 139.5 | 186.5 |  | 1.00±0.00 | 0 | 0 |  | 4.00±0.00 | 0 |
| #20 | UCB1 | 1900 | 1815 | 110 | 92.5 | 42.5 | 2.23 | 0.86 | 0.62 | 0.083 | 0.0025 | 0.025 | 0.0025 | 102.30 | 136.76 |  | 1.57±0.20 | 3.00±0.37 | 0.57±0.10 |  | 2.92±0.51 | 0.056±0.007 |
| #21 | UCB1 | 950 | 1072.5 | 715 | 601.25 | 276.25 | 45.715 | 17.63 | 12.71 | 1.7015 | 0.05125 | 0.5125 | 0.05125 | 83.7 | 111.9 |  | 3.05±0.17 | 2.85±0.22 | 0.88±0.08 |  | 1.32±0.16 | 0.184±0.017 |
| #22 | UCB1 | 1900 | 1815 | 1320 | 1110 | 510 | 60.21 | 23.22 | 16.74 | 2.241 | 0.0675 | 0.675 | 0.0675 | 139.5 | 186.5 |  | 1.00±0.00 | 0 | 0 |  | 4.00±0.00 | 0 |
| #23 | UCB1 | 1900 | 330 | 1320 | 1110 | 510 | 60.21 | 23.22 | 16.74 | 2.241 | 0.0675 | 0.675 | 0.0675 | 139.5 | 186.5 |  | 1.00±0.00 | 0 | 0 |  | 4.00±0.00 | 0 |
| #24 | UCB1 | 950 | 1072.5 | 715 | 601.25 | 276.25 | 45.715 | 17.63 | 12.71 | 1.7015 | 0.05125 | 0.5125 | 0.05125 | 55.8 | 74.6 |  | 3.28±0.21 | 2.78±0.19 | 1.15±0.15 |  | 1.05±0.05 | 0.229±0.016 |
| #25 | UCB1 | 1266.66 | 330 | 1320 | 1110 | 510 | 31.22 | 12.04 | 8.68 | 1.162 | 0.035 | 0.35 | 0.035 | 27.9 | 37.3 |  | 2.50±0.13 | 3.36±0.36 | 0.71±0.07 |  | 1.13±0.13 | 0.211±0.025 |
| #26 | UCB1 | 0 | 1320 | 1320 | 1110 | 510 | 31.22 | 12.04 | 8.68 | 1.162 | 0.035 | 0.35 | 0.035 | 139.5 | 186.5 |  | 2.20±0.21 | 3.30±0.33 | 0.45±0.06 |  | 1.35±0.23 | 0.059±0.004 |
| #27 | UCB1 | 0 | 1320 | 110 | 92.5 | 42.5 | 89.2 | 34.4 | 24.8 | 3.32 | 0.1 | 1 | 0.1 | 65.09 | 87.03 |  | 2.17±0.27 | 2.33±0.40 | 0.61±0.05 |  | 2.11±0.45 | 0.056±0.007 |
| #28 | UCB1 | 633.33 | 1815 | 513.33 | 431.66 | 198.33 | 89.2 | 34.4 | 24.8 | 3.32 | 0.1 | 1 | 0.1 | 139.5 | 186.5 |  | 2.63±0.20 | 2.75±0.30 | 0.70±0.08 |  | 1.05±0.80 | 0.115±0.011 |
| MS (Control) | UCB1 | 1900 | 1650 | 440 | 370 | 170 | 22.3 | 8.6 | 6.2 | 0.83 | 0.025 | 0.25 | 0.025 | 27.9 | 37.3 |  | 3.00±0.35 | 2.60±0.50 | 2.36±0.70 |  | 2.10±0.55 | 0.225±0.030 |
| #1 | Ghazvini | 1900 | 330 | 110 | 92.5 | 42.5 | 89.2 | 34.4 | 24.8 | 3.32 | 0.1 | 1 | 0.1 | 65.09 | 87.03 |  | 2.28±0.14 | 2.89±0.38 | 0.75±0.12 |  | 1.88±0.18 | 0.084±0.009 |
| #2 | Ghazvini | 0 | 330 | 916.66 | 770.83 | 354.16 | 89.2 | 34.4 | 24.8 | 3.32 | 0.1 | 1 | 0.1 | 27.9 | 37.3 |  | 2.62±0.12 | 3.45±0.24 | 0.60±0.02 |  | 1.00±0.00 | 0.191±0.012 |
| #3 | Ghazvini | 1900 | 1815 | 916.66 | 770.83 | 354.16 | 2.23 | 0.86 | 0.62 | 0.083 | 0.0025 | 0.025 | 0.0025 | 27.9 | 37.3 |  | 1.96±0.25 | 3.18±0.32 | 0.83±0.07 |  | 1.28±0.14 | 0.187±0.018 |
| #4 | Ghazvini | 0 | 1815 | 110 | 92.5 | 42.5 | 31.22 | 12.04 | 8.68 | 1.162 | 0.035 | 0.35 | 0.035 | 102.30 | 136.76 |  | 1.25±0.08 | 2.20±0.24 | 0.70±0.09 |  | 3.65±0.15 | 0.001±0.001 |
| #5 | Ghazvini | 0 | 1815 | 1320 | 1110 | 510 | 89.2 | 34.4 | 24.8 | 3.32 | 0.1 | 1 | 0.1 | 27.9 | 37.3 |  | 3.60±0.14 | 3.40±0.42 | 1.36±0.14 |  | 1.75±0.38 | 0.287±0.047 |
| #6 | Ghazvini | 950 | 330 | 1320 | 1110 | 510 | 2.23 | 0.86 | 0.62 | 0.083 | 0.0025 | 0.025 | 0.0025 | 139.5 | 186.5 |  | 1.00±0.00 | 0 | 0 |  | 4.00±0.00 | 0 |
| #7 | Ghazvini | 0 | 330 | 110 | 92.5 | 42.5 | 2.23 | 0.86 | 0.62 | 0.083 | 0.0025 | 0.025 | 0.0025 | 27.9 | 37.3 |  | 1.95±0.24 | 4.10±0.43 | 0.51±0.04 |  | 2.38±0.46 | 0.033±0.007 |
| #8 | Ghazvini | 0 | 1815 | 1320 | 1110 | 510 | 89.2 | 34.4 | 24.8 | 3.32 | 0.1 | 1 | 0.1 | 139.5 | 186.5 |  | 1.89±0.20 | 3.44±0.05 | 0.73±0.04 |  | 1.88±0.45 | 0.060±0.017 |
| #9 | Ghazvini | 633.33 | 825 | 110 | 92.5 | 42.5 | 2.23 | 0.86 | 0.62 | 0.083 | 0.0025 | 0.025 | 0.0025 | 139.5 | 186.5 |  | 1.00±0.00 | 0 | 0 |  | 4.00±0.00 | 0 |
| #10 | Ghazvini | 0 | 825 | 1320 | 1110 | 510 | 2.23 | 0.86 | 0.62 | 0.083 | 0.0025 | 0.025 | 0.0025 | 65.09 | 87.03 |  | 2.50±0.17 | 5.45±0.45 | 0.52±0.05 |  | 1.18±0.18 | 0.146±0.019 |
| #11 | Ghazvini | 1900 | 1815 | 110 | 92.5 | 42.5 | 89.2 | 34.4 | 24.8 | 3.32 | 0.1 | 1 | 0.1 | 27.9 | 37.3 |  | 1.70±0.18 | 3.00±0.25 | 0.79±0.08 |  | 2.95±0.31 | 0.102±0.006 |
| #12 | Ghazvini | 0 | 330 | 513.33 | 431.66 | 198.33 | 60.21 | 23.22 | 16.74 | 2.241 | 0.0675 | 0.675 | 0.0675 | 139.5 | 186.5 |  | 1.11±0.07 | 3.33±0.47 | 0.64±0.03 |  | 3.87±0.08 | 0.026±0.004 |
| #13 | Ghazvini | 1900 | 1072.5 | 110 | 92.5 | 42.5 | 2.23 | 0.86 | 0.62 | 0.083 | 0.0025 | 0.025 | 0.0025 | 27.9 | 37.3 |  | 1.90±0.20 | 3.40±0.54 | 0.81±0.11 |  | 2.61±0.37 | 0.086±0.011 |
| #14 | Ghazvini | 633.33 | 330 | 1320 | 1110 | 510 | 89.2 | 34.4 | 24.8 | 3.32 | 0.1 | 1 | 0.1 | 102.30 | 136.76 |  | 2.19±0.04 | 2.63±0.26 | 0.94±0.08 |  | 1.28±0.28 | 0.076±0.018 |
| #15 | Ghazvini | 1900 | 330 | 513.33 | 431.66 | 198.33 | 2.23 | 0.86 | 0.62 | 0.083 | 0.0025 | 0.025 | 0.0025 | 102.30 | 136.76 |  | 1.58±0.16 | 2.65±0.31 | 0.67±0.05 |  | 2.76±0.32 | 0.052±0.007 |
| #16 | Ghazvini | 1266.66 | 1815 | 1320 | 1110 | 510 | 2.23 | 0.86 | 0.62 | 0.083 | 0.0025 | 0.025 | 0.0025 | 102.30 | 136.76 |  | 1.56±0.29 | 1.88±0.22 | 0.82±0.18 |  | 2.91±0.41 | 0.037±0.005 |
| #17 | Ghazvini | 0 | 1815 | 715 | 601.25 | 276.25 | 2.23 | 0.86 | 0.62 | 0.083 | 0.0025 | 0.025 | 0.0025 | 27.9 | 37.3 |  | 2.50±0.20 | 3.00±0.48 | 0.58±0.06 |  | 1.00±0.00 | 0.227±0.022 |
| #18 | Ghazvini | 1900 | 1320 | 1320 | 1110 | 510 | 89.2 | 34.4 | 24.8 | 3.32 | 0.1 | 1 | 0.1 | 65.09 | 87.03 |  | 2.80±0.24 | 3.00±0.29 | 0.99±0.23 |  | 1.35±0.19 | 0.228±0.048 |
| #19 | Ghazvini | 1900 | 1320 | 110 | 92.5 | 42.5 | 60.21 | 23.22 | 16.74 | 2.241 | 0.0675 | 0.675 | 0.0675 | 139.5 | 186.5 |  | 1.00±0.00 | 0 | 0 |  | 4.00±0.00 | 0 |
| #20 | Ghazvini | 1900 | 1815 | 110 | 92.5 | 42.5 | 2.23 | 0.86 | 0.62 | 0.083 | 0.0025 | 0.025 | 0.0025 | 102.30 | 136.76 |  | 1.22±0.12 | 2.00±0.33 | 0.76±0.10 |  | 3.92±0.07 | 0.023±0.006 |
| #21 | Ghazvini | 950 | 1072.5 | 715 | 601.25 | 276.25 | 45.715 | 17.63 | 12.71 | 1.7015 | 0.05125 | 0.5125 | 0.05125 | 83.7 | 111.9 |  | 2.63±0.24 | 2.80±0.31 | 1.02± 0.09 |  | 1.81±0.25 | 0.088±0.010 |
| #22 | Ghazvini | 1900 | 1815 | 1320 | 1110 | 510 | 60.21 | 23.22 | 16.74 | 2.241 | 0.0675 | 0.675 | 0.0675 | 139.5 | 186.5 |  | 1.00±0.00 | 0 | 0 |  | 4.00±0.00 | 0 |
| #23 | Ghazvini | 1900 | 330 | 1320 | 1110 | 510 | 60.21 | 23.22 | 16.74 | 2.241 | 0.0675 | 0.675 | 0.0675 | 139.5 | 186.5 |  | 1.00±0.00 | 0 | 0 |  | 4.00±0.00 | 0 |
| #24 | Ghazvini | 950 | 1072.5 | 715 | 601.25 | 276.25 | 45.715 | 17.63 | 12.71 | 1.7015 | 0.05125 | 0.5125 | 0.05125 | 55.8 | 74.6 |  | 3.80±0.06 | 2.55±0.23 | 1.35±0.11 |  | 1.12±0.08 | 0.182±0.014 |
| #25 | Ghazvini | 1266.66 | 330 | 1320 | 1110 | 510 | 31.22 | 12.04 | 8.68 | 1.162 | 0.035 | 0.35 | 0.035 | 27.9 | 37.3 |  | 3.14±0.21 | 2.86±0.63 | 0.86±0.10 |  | 1.14±0.14 | 0.183±0.007 |
| #26 | Ghazvini | 0 | 1320 | 1320 | 1110 | 510 | 31.22 | 12.04 | 8.68 | 1.162 | 0.035 | 0.35 | 0.035 | 139.5 | 186.5 |  | 1.50±0.21 | 3.30±0.53 | 0.59±0.05 |  | 2.37±0.53 | 0.039±0.005 |
| #27 | Ghazvini | 0 | 1320 | 110 | 92.5 | 42.5 | 89.2 | 34.4 | 24.8 | 3.32 | 0.1 | 1 | 0.1 | 65.09 | 87.033 |  | 1.86±0.09 | 2.55±0.34 | 0.85±0.12 |  | 1.61±0.33 | 0.039±0.012 |
| #28 | Ghazvini | 633.33 | 1815 | 513.33 | 431.66 | 198.33 | 89.2 | 34.4 | 24.8 | 3.32 | 0.1 | 1 | 0.1 | 139.5 | 186.5 |  | 1.30±0.13 | 2.00±0.25 | 0.96±0.10 |  | 4.00±0.00 | 0.046±0.008 |
| MS (Control) | Ghazvini | 1900 | 1650 | 440 | 370 | 170 | 22.3 | 8.6 | 6.2 | 0.83 | 0.025 | 0.25 | 0.025 | 27.9 | 37.3 |  | 3.17±0.27 | 3.33±0.42 | 1.60±0.14 |  | 2.83±0.38 | 0.255±0.021 |
