# Supplementary material for: Combining DOE With Neurofuzzy Logic for Healthy Mineral Nutrition of Pistachio Rootstocks in vitro Culture
Source: Front Plant Sci. 2018 Oct 15;9:1474. doi: 10.3389/fpls.2018.01474 (PMC6196285; doi:10.3389/fpls.2018.01474)
Supplement: Supplementary file 4 [file Table_2.DOC]

**TABLE S2|** Rule generated by neurofuzzy logic model with membership degrees less than 1.00.

| Rules |  | Genotype | NH_4_^+^ | NO_3_^-^ | K^+^ | SO_4_^2-^ | Fe^2+^ | EDTA^-^ | Cu^2+^ | Cl^-^ | BO_3_^-^ |  | SQ | PR | SL (cm) | STN | BC  (g) | Membership degree |
| --- | --- | --- | --- | --- | --- | --- | --- | --- | --- | --- | --- | --- | --- | --- | --- | --- | --- | --- |
| 1 | IF |  |  |  |  | Low |  |  |  | Low |  | THEN | Low |  |  |  |  | 0.74 |
| 2 |  |  |  |  |  | Low |  |  |  | High |  |  | Low |  |  |  |  | 0.65 |
| 4 |  |  |  |  |  | Mid |  |  |  | High |  |  | Low |  |  |  |  | 0.74 |
| 9 |  |  |  |  | Low |  |  |  |  |  |  |  | Low |  |  |  |  | 0.76 |
| 12 |  |  | Mid |  |  |  |  |  |  |  |  |  | Low |  |  |  |  | 0.56 |
| 14 | IF |  |  |  | Low | Low |  | Low |  |  |  | THEN |  | High |  |  |  | 0.62 |
| 15 |  |  |  |  | Low | High |  | Low |  |  |  |  |  | High |  |  |  | 0.73 |
| 16 |  |  |  |  | High | Low |  | Low |  |  |  |  |  | High |  |  |  | 0.59 |
| 17 |  |  |  |  | High | High |  | Low |  |  |  |  |  | High |  |  |  | 0.70 |
| 22 |  |  |  |  |  |  | Low |  |  |  | Low |  |  | High |  |  |  | 0.50 |
| 23 |  |  |  |  |  |  | Low |  |  |  | High |  |  | Low |  |  |  | 0.55 |
| 25 |  |  |  |  |  |  | Mid |  |  |  | High |  |  | High |  |  |  | 0.53 |
| 26 |  |  |  |  |  |  | High |  |  |  | Low |  |  | Low |  |  |  | 0.99 |
| 27 |  |  |  |  |  |  | High |  |  |  | High |  |  | High |  |  |  | 0.56 |
| 30 | IF |  |  | High |  |  | Low |  | Low |  |  | THEN |  |  | Low |  |  | 0.67 |
| 35 |  |  |  | Mid |  |  | Low |  | High |  |  |  |  |  | High |  |  | 0.56 |
| 36 |  |  |  | High |  |  | Low |  | High |  |  |  |  |  | Low |  |  | 0.76 |
| 38 |  |  |  | Mid |  |  | High |  | Low |  |  |  |  |  | Low |  |  | 0.74 |
| 39 |  |  |  | High |  |  | High |  | Low |  |  |  |  |  | Low |  |  | 0.96 |
| 43 |  |  |  | Low |  |  | High |  | High |  |  |  |  |  | Low |  |  | 0.68 |
| 46 |  |  |  |  | Low |  |  |  |  | Low |  |  |  |  | Low |  |  | 0.78 |
| 48 |  |  |  |  | High |  |  |  |  | Low |  |  |  |  | Low |  |  | 0.69 |
| 49 |  |  |  |  | Low |  |  |  |  | High |  |  |  |  | High |  |  | 0.71 |
| 50 |  |  |  |  | Mid |  |  |  |  | High |  |  |  |  | Low |  |  | 0.72 |
| 51 |  |  |  |  | High |  |  |  |  | High |  |  |  |  | Low |  |  | 0.95 |
| 52 |  | UCB1 |  |  |  |  |  |  |  |  |  |  |  |  | High |  |  | 0.56 |
| 53 |  | Ghazvini |  |  |  |  |  |  |  |  |  |  |  |  | High |  |  | 0.63 |
| 60 | IF |  |  |  | High |  |  |  |  |  |  | THEN |  |  |  | High |  | 0.95 |
| 61 |  | UCB1 |  |  |  |  |  |  |  |  |  |  |  |  |  | Low |  | 0.87 |
| 62 |  | Ghazvini |  |  |  |  |  |  |  |  |  |  |  |  |  | High |  | 0.66 |
| **63** | IF |  |  |  |  |  | **Low** |  |  |  |  | THEN |  |  |  |  | **High** | **0.97** |
| 66 |  |  |  |  |  | Mid |  |  |  |  |  |  |  |  |  |  | High | 0.58 |
| 67 |  |  |  |  |  | High |  |  |  |  |  |  |  |  |  |  | High | 0.52 |
